# Strain modulating electronic band gaps and SQ efficiencies of semiconductor 2D PdQ_2_ (Q = S, Se) monolayer

**DOI:** 10.1038/s41598-022-06142-6

**Published:** 2022-02-22

**Authors:** Dhara Raval, Sanjeev K. Gupta, P. N. Gajjar, Rajeev Ahuja

**Affiliations:** 1 Department of Physics, University School of Sciences, Gujarat University, Ahmedabad, 380009 India; 2 Computational Materials and Nanoscience Group, Department of Physics and Electronics, St. Xavier’s College, Ahmedabad, 380009 India; 3 Condensed Matter Theory Group, Department of Physics and Astronomy, Uppsala University, Box 516, 751 20 Uppsala, Sweden; 4 Department of Physics, Indian Institute of Technology Ropar, Rupnagar, Punjab 140001 India

**Keywords:** Two-dimensional materials, Electronic devices

## Abstract

We studied the physical, electronic transport and optical properties of a unique pentagonal PdQ_2_ (Q = S, Se) monolayers. The dynamic stability of 2Dwrinkle like-PdQ_2_ is proven by positive phonon frequencies in the phonon dispersion curve. The optimized structural parameters of wrinkled pentagonal PdQ_2_ are in good agreement with the available experimental results. The ultimate tensile strength (UTHS) was calculated and found that, penta-PdS_2_ monolayer can withstand up to 16% (18%) strain along *x* (*y*) direction with 3.44 GPa (3.43 GPa). While, penta-PdSe_2_ monolayer can withstand up to 17% (19%) strain along *x* (*y*) dirrection with 3.46 GPa (3.40 GPa). It is found that, the penta-PdQ_2_ monolayers has the semiconducting behavior with indirect band gap of 0.94 and 1.26 eV for 2D-PdS_2_ and 2D-PdSe_2_, respectively. More interestingly, at room temperacture, the hole mobilty (electron mobility) obtained for 2D-PdS_2_ and PdSe_2_ are 67.43 (258.06) cm^2^ V^−1^ s^−1^ and 1518.81 (442.49) cm^2^ V^−1^ s^−1^, respectively. In addition, I-V characteristics of PdSe_2_ monolayer show strong negative differential conductance (NDC) region near the 3.57 V. The Shockly-Queisser (SQ) effeciency prameters of PdQ_2_ monolayers are also explored and the highest SQ efficeinciy obtained for PdS_2_ is 33.93% at −5% strain and for PdSe_2_ is 33.94% at −2% strain. The penta-PdQ_2_ exhibits high optical absorption intensity in the UV region, up to 4.04 × 10^5^ (for PdS_2_) and 5.28 × 10^5^ (for PdSe_2_), which is suitable for applications in optoelectronic devices. Thus, the ultrathin PdQ_2_ monolayers could be potential material for next-generation solar-cell applications and high performance nanodevices.

## Introduction

Atomically thin two-dimensional (2D) structures like, honeycomb lattice graphene^1,2^, boron nitride (BN)^3,4^, transition metal dichalcogenides (TMDs), group—IV, V^5,6^ elements has recived attention due to their potential applications in the field of electronics, optoelectronics, solar cell, energy harvesting, and biomedical technologies with the high possibilities of replacing traditional 2D materials^1–7^. Among the 2D materials, if targeting application in nanodevices, MX_2_ (M = Mo, W, V, Ti, Pd, Pt; X = S, Se, Te) type TMDs are technologically favourable materials^7–9^. Nowadays, an endless research is going on traditional TMDs layered materials, which is in demand of high mobility, high stability in air and strain modulated bandgap. Of these, many of TMDs has been already synthesized via the various method of exfoliation^10–14^. Interestingly, palladium based dichalcogenides has brought 2D with buckled or puckered pentagonal structures under the spotlight because of its unique lattice arrangement and fascinating properties such as fine tuning their physical, elctronic, thermal and optical properties.

Gronvold et al., studied the PdS_2_/PtS_2_ and concluded that noble metals (Pd and Pt) would be a layered structure with sulphur atoms^15,16^. Afterwards, in 2015 Wang et al., theoretically reported that 2D PdS_2_ is semiconductor with moderate indirect band gap up to ~ 1.60 eV, which is very significant in fabrication of nanodevices^17^. Moreover, Cheng et al.,^18^ have developed ultrafast Yb doped fiber laser using few layers of PdS_2_ and indicated that layered PdS_2_ can be a favourable candidate for photonics application. Some work have been reported on the layered PdSe_2_ via method of mechanical exfoliated, atomic-resolution scanning transmission electron microscopy (AR-STEM) and chemical vapor deposition (CVD)^14,19–24^. For Example, Akinola et al.,^24^ had fabricated few layers puckered pentagonal PdSe_2_ by AR-STEM method and demonstrated that, PdSe_2_ exhibits good ambipolar semiconducting nature with room temperature electron-apparent field-effect mobility (up to ~ 158 cm^2^ V^−1^ s^−1^) and its stability is remain same up to 60 days. It has been noted that having high mobility (electron/hole) and band gap between 1.2 ~ 1.9 eV of TMD, it makes them suitable for the FETs (field effect transistors) and CMOS applications, respectively^25–28^.

Recently, Weiting and their research group^29^ had successfully synthesized 2D PdSe_2_ nanosheets on 300 nm SiO_2_/Si substract and claimed that it is really promising material to make infrared photodetector due to its high-photoresponsivity (~ 660 A.W^−1^ under 914 nm laser) in harsh condition, which may be benefitted in military field for night-time detection because of its ability to work at night as well as in bad whether conditions. Beside these, few other reports theoretical are also available on the 2D pentagonal type PdQ_2_ monolayers^17,30–34^. Deng et al.,^31^ investigated the strain applied mechanical, electronic and optical properties of PdS_2_, PdP_2_ and PtSe_2_. Except this, none of works have been reported on strain applied mechanical, electronic, optical and electronic transport properties of 2D penta-PdQ_2_ (Q = S, Se). Further, the lower band gap of 2D-PdQ_2_ monolayers has also motivated us to investigate absorbance of solar radiation on nanosheet. Hence, we also studied the strain dependent solar cell power conversion efficiency % (PCE) of penta-PdQ_2_ (Q = S, Se) monolayers.

In this work, we have investigated the geometric, mechanical, electronic transport and optical properties of penta-PdQ_2_ using density functional theory (DFT). After the optimization of structures, we analysed the lattice parameters (Å), band gap (eV) and effective mass (m*). Then after we applied biaxial strain ɛ (%) and obtained its bulk modulus *B* (*GPa*). The ultimate tensile strength (UTHS) is also found under the tensile strain (+ ɛ%) for PdQ_2_ (Q = S, Se). We also studied the influence of loading on the band edges and obtained carrier mobility (µ_2D_) along biaxial strain ɛ (%). We have adopted Shockley-Queisser (SQ) method to study Solar cell efficiency η (%) and calculated the maximum power density (${P}_{max}$). The optical properties including the dielectric constants, absorption co-efficient, refractive index, and reflectivity of penta-PdQ_2_ (Q = S, Se) monolayers are also reported in this paper. Herein, "Computational methods" section contains the methedology of computations, "Results and discussion" section includes the results and disccusion part of the work on penta-PdQ_2_ (Q = S, Se) monolayers.

## Computational methods

**Figure 1:**
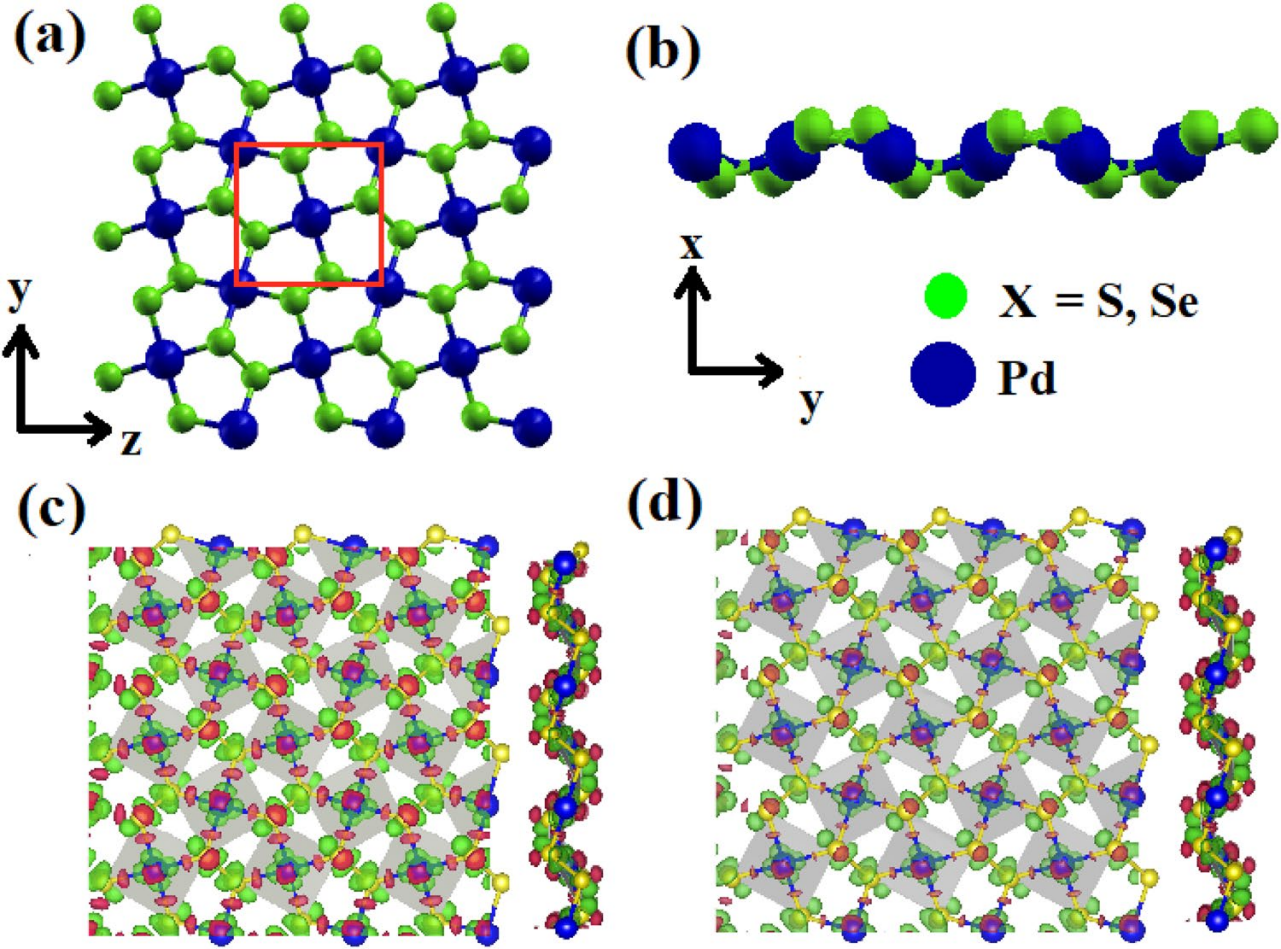
(**a**) Top-view of penta-PdQ_2_ (Q = S, Se) monolayer structure. Red line indicats the unitcell of the system. (**b**) Side views from *z-* direction of the atomic structure of penta-PdQ_2_. The navy blue and light-green circles represent the Pd and Q (S, Se) atoms, respectively. The deformation charge density plot (**c**) for penta-PdS_2_ (**d**) for penta-PdSe_2_; where red and green colour indicate the charge accumulation (electron excess) and charge deplation (electron loss). The iso-level is 0.063 *e/Å*^*3*^ for both monolayers.

The structural, electronic transport and optical properties of 2D wrinkled PdQ_2_ (Q = S, Se) monolayers were performed with in the SIESTA code^35^. The exchange–correlation functional approach was used in term of Perdew–Burke-Ernzerhof (PBE) type pseudopotential^36^. The basic unit cell of 2D-PdQ_2_ (Q = S, Se) contains two Pd atoms and four Q (Q = S, Se) atoms as shown in Fig. 1. An energy cut-off of 450 and 300 *Ry* for PdS_2_ and PdSe_2_ monolayers were adopted, respectively. The doubled zeta plus (DZP) basis set was used with an energy of 0.02 *Ry* to expand the Kohn–Sham orbital. The Г- centred mesh of 20 × 20 × 1 and 15 × 15 × 1 k-points were sampled for PdS_2_ and PdSe_2_ monolayers, respectively under the Monkhorst–pack scheme in the 2D Brillouin zone^37^. The force 0.01 eV/Å was kept to relaxing the unstrained and strained structures of penta-PdQ_2_ (Q = S, Se) monolayers. The phonon dispersive curves were calculated using the density functional perturbation theory (DFPT) formalism^38^. In all calculation, the Fermi level (*E*_*F*_) is shifted at the zero energy. The parameters of carrier mobility of penta-PdQ_2_ (Q = S, Se) monolayers were found such as i.e., effective mass (m*), stiffness constants (*C*_*2D*_) and deformation energy (*E*_*1*_) approximations using the following formula proposed by the Bardeen and Shockley^39^, *µ*_*2D*_ = $\frac{2e{\hbar }^{3}{C}_{2D}}{{3k}_{B}T{{|{m}^{*}|}^{2}\left({E}_{1}\right)}^{2}}$ ; here, *e* is elementary charge of an electron, *ђ* is the reduced Planck’s constant, *C*_*2D*_ is the in-plane stiffness constant and for 2D system it is defined as ${C}_{2D}$ = $\frac{1}{{S}_{0}}\frac{{\partial }^{2}E}{\partial {(\frac{a}{{a}_{0}})}^{2}}$ ; where *S*_*0*_ is cell area and *a*_*0*_ is the lattice constant at equilibrium, *E* and *a* are the total energy and lattice constant of the monolayer after deformation, ${k}_{B}$ is the Boltzmann constant, *T* is the temperature (*300 K*). Also, m* is the effective mass in the transport direction and E_1_ is the deformation potential (DP) constant denoting the shifting of each band edges due to the applied biaxial strain *ε*_*xy*_ defined as ${E}_{1}$= $\frac{\partial {E}_{edge}}{\partial (\frac{a}{{a}_{0}})}$; where *E*_*edge*_ is energy value of VBM (for holes) and CBM (for electrons).

The electronic transport properties of PdQ_2_ monolayers are calculated by utilizing the non-equilibrium Green’s function (NEGF) in the form of *I-V* response by TRANSIESTA module of SIESTA code^40,41^. The electric current through the scattering region is calculated by exploring the Landauer-Buttiker formalism,

1 $$I\left({V}_{b}\right)={G}_{0}{\int }_{{\mu }_{R}}^{{\mu }_{L}} T\left(E,{V}_{b}\right) \left[f \left(\mathrm{\rm E}-{\mu }_{L}\right)-f \left(\mathrm{\rm E}-{\mu }_{R}\right)\right] dE$$

where T (E, V_bias_) is the transmission probability of an electron incident at an energy E through the device under the bias voltage V_bias_, ${\mu }_{L}$ and ${\mu }_{R}$ are the electrostatic potentials of left and right electrodes at a particular voltage bias and G_0_ is the unit of quantum conductance. The optical properties were investigated including the dielectric function and absorbance coefficient by DFT within the random phase approximation (RPA)^42^. The dielectric function is given by *ɛ (ω)* = *ɛ’ (ω)* + *i ε’’ (ω)*. Here, *ε’(ω)* and *ε’’(ω)* are the real and imaginary parts of the complex dielectric function^43^, respectively.

## Results and discussion

### Stability and mechanical properties

First, the geometric structures of pristine 2D penta-PdQ_2_ (Q = S, Se) were systemitically relaxed and lattice structures of penta- PdQ_2_ were obtained. It is built up with two Pd and four Q (S, Se) atoms in the unitcell as shown in Fig. 1a,b. The differential charge density in Fig. 1c,d shows an accumulation and depletion of charges between Pd and Q (S, Se) atoms. In Table 1, the relaxed structural parameters are summarized with ground state properties such as, lattice parameter (*Å*), distance d (*Å*) between Pd-Q_1_, Pd-Q_2_ and Q_1_-Q_2_, thickness *t* (*Å*) and cohesive energy *E*_*coh*_ (eV/atom) of PdQ_2_ monolayers.

**Table 1:** The calculated lattice parameter (Å), bond lengths d (Å), thickness t (Å) and cohesive energy *E*_*coh*_ (eV/atom) of penta-PdQ_2_ monolayers.

Penta-PdQ_2_ (Q = S, Se)		d_Pd-Q1_ (Å)	d_Pd-Q2_ (Å)	d_X1-Q2_ (Å)	Thickness (Å)	Lattice parameter (Å)	Cohesive energy Ecoh (eV/atom)
Penta-PdS_2_	Present work	2.35	2.34	2.14	1.29	a = 5.62, b = 5.42	−4.71
	Other DFT/ *Exp	2.34^17^ 2.35^32^	2.35^17^ 2.34^32^	2.10^17^ 2.11^32^	1.28^32^	a = 5.49, b = 5.59^17^ a = 5.48, b = 5.59^32^ a = 5.48, b = 5.57^33^	–
Penta- PdSe_2_	present work	2.45	2.45	2.42	1.47	a = 5.94, b = 5.68	−4.30
	Other DFT/ *Exp	2.45^34^	2.46^34^	2.42^34^	–	a = 5.72, b = 5.80^29^ * a = 5.74, b = 5.91^32^ a = 5.75, b = 5.92^33^ a = 5.71, b = 5.90^34^	–

As shown, in Table 1, the obtained lattice parameters *a* (*b*) of PdS_2_ and PdSe_2_ monolayers are 5.62 (5.42) Å and 5.94 (5.68) Å, respectively. The atomic distance between Pd-S_1_(Se_1_), Pd-S_2_(Se_2_), and S_1_(Se_1_)-S_2_(Se_2_) atoms are 2.35 (2.45) Å, 2.34 (2.42) Å and 2.14 (2.42) Å, respectively and shows good agreement with previously reported results^17,29,32–34^. To evaluate stability of resulted structures, the cohesive energy is calculated using the formula: *E*_*coh*_ = *Σ n*_*X*_* E*_*X*_*—E*_*PdQ2*_*/N*. where *E*_*PdQ2*_ denotes total energy of PdQ_2_ (Q = S, Se) unit cell, *N* is total atom in unit cell and *n*_*X*_ and *E*_*X*_ are number of atoms of each element and atomic energies of each atom in the unit cell, respectively. It is found that cohesive energies for PdS_2_ and PdSe_2_ monolayer are −4.70 and −4.30 eV*/atom*, respectively. Although this is larger than that for the 2D-antimony (−4.03), 2D-arsenene (−2.96), 2D-MoS_2_ (−4.11), 2D-MoSe_2_ (−3.94) and 2D-black phosphorous (−3.48). This is evidence that penta-PdQ_2_ monolayers have strong bonding in its ring-network. In addition, to confirm the kinetic stability of penta-PdQ_2_ (Q = S, Se) structures, we calculated the phonon dispersion curves along high symmetric points for both PdS_2_ and PdSe_2_ monolayers and results are as shown in Figure S1 (a, b), ESI^†^. The highest phonon frequencies extended is up to 2275 cm^-1^ and 2193 cm^-1^ for PdS_2_ and PdSe_2_ monolayers, respectively. Evidently, no negative acoustics branch is appeared in the first Brillouin zone, itself proves the dynamical stability of both the monolayers at room temperature.

Next, we focused on mechanical properties of penta-PdQ_2_ and evaluated Young’s modulus *Y* (N/m) and bulk modulus *B* (N/m). The Young’s moduli *Y* (N/m) and bulk moduli *B* (N/m) were investigated under uniaxial and biaxial strain on the penta-PdQ_2_ monolayers. The range of applied strain $\varepsilon (\mathrm{\%})$ is taken in interval of −5 $\mathrm{\%}$ $\le$ $\varepsilon$ $\le$ +5 $\mathrm{\%}$ in the step of 1%. The bulk modulus computed by applying biaxial load (*x* and *y*) on the penta-PdQ_2_ monolayer with following relation^44,45^,

2 $$B =\mathrm{S}0\frac{{\partial }^{2}{E}_{s}}{\partial {A}^{2}}$$

where *S*_*0*_ is unstrained cross-sectional area of the unit cell. *E*_*s*_ and *A* are the total strain energy and area of respected applied strain, respectively. Figure S2 (a, b), ESI^†^ shows the strain energy vs area curve for pristine and loaded structures of PdS_2_ and PdSe_2_ monolayers, respectively. This curve is fitted by the polynomial curve fitting and with help of Eq. (1), we have obtained bulk modulus *B* (N/m) as shown in Figure S2a,b, ESI^†^. The calculated bulk modulus (N/m) of PdS_2_ and PdSe_2_ are 30.22 and 23.56 N/m, respectively. These values are larger than that for the monolayer of Sb (~ 21.88 N*/m*) and As (~ 25.78 N*/*m)^44,46^, that indicate that penta-PdQ_2_ has stiffer and better resistance to deformation compared to antimony (Sb) and arsenene (As) monolayers. Afterwards, we have calculated the in-plane Young’s modulus *Y* (N/m) in longitudinal (along *x*-axis) or transverse (along *y*-axis) direction for penta-PdQ_2_. To obtained Young’s modulus from first-principles calculation, the following formula was employed^44,45^,

3 $$Y =\frac{1}{{S}_{0}}\frac{{\partial }^{2}{E}_{s}}{\partial {\varepsilon }^{2}}$$

Here, *S*_*0*_ is unstrained cross-sectional area of the unicell. Next, $\frac{{\partial }^{2}{E}_{s}}{\partial {\varepsilon }^{2}}$ shows the second derivative of strain energy (eV) with applied load. Here, the compressive and tensile strains are applied to the penta-PdQ_2_ in the longitudinal or transverse direction. The Young’s moduli obtained by using the curves shown in Figure S3a,b, ESI^†^. The calculated Young’s modulus Y (N/m) along *x* (*y*) direction are 74.13 (42.40) N/m and 65.26 (28.62) N/m for penta-PdS_2_ and penta-PdSe_2_, respectively. The results indicating that, Young’s modulus decreases as we switched S to Se, and this is due to the increment in the Pd-S and Pd–Se bond strength. Xiong et al.^47^ had calculated orientation-dependent Young’s modulus of 2D orthorhombic MX_2_ (M = Ni, Pd; X = S, Se, Te) and reported values are ~ 50 (37) *N/m* for PdS_2_ (PdSe_2_). From the present investigation of Young’s modulus of penta-PdQ_2_, we suggest that the 2D penta-PdQ_2_ can be preferred candidates for flexible devices because of having ultra-low values of Young’s modulus, compared to that of 2D graphene (340 N/m) and 2D MoS_2_ (125 N/m)^48,49^.

**Figure 2:**
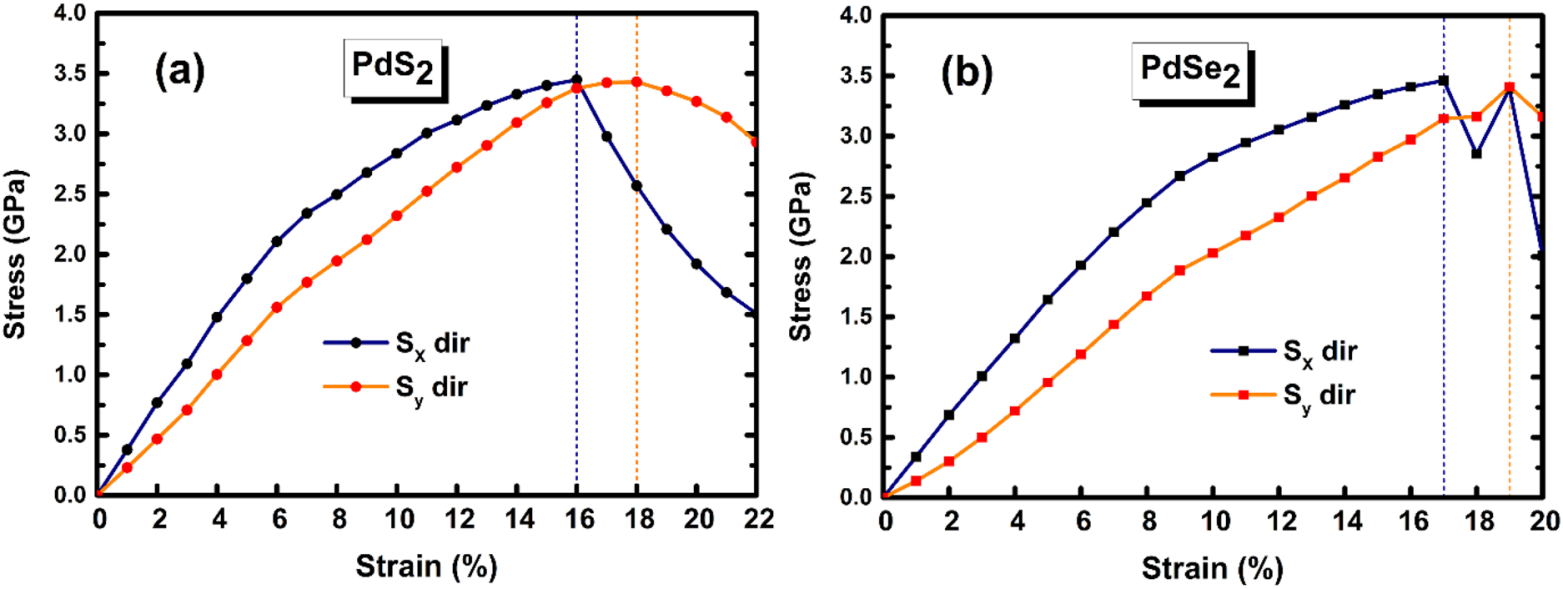
The stress (GPa) vs strain ($\mathrm{\%}$) curve under tensile strain. (**a**) For penta-PdS_2_ and (**b**) For penta- PdSe_2_.

Further, we have also explored the mechanical stability of penta-PdQ_2_ monolayers and examined that up to which values of strain ($\mathrm{\%}$) the monolayers can withstand? It is worth to find curve of stress–strain relation called ultimate tensile strength (UTSH) curve^50^. The UTSH is representing the maximum stress value (*GPa*) that a monolayer can withstand prior to the fractured structure set. This can be calculated by the components of stress tensor with respect applied strain. The computed UTSH for penta-PdQ_2_ monolayers are shown in Fig. 2a,b. The stress value, where the slope of the stress–strain curve becomes zero indicates the value of UTSH, and the strain at this value represents the magnitude of ultimate tensile strain (UTSR). In case of penta-PdS_2_, the calculated values of UTSR along *x* (*y*) directions is 16$\mathrm{\%}$ (18$\mathrm{\%}$) at stress of 3.44 *GPa* and 3.43 *GPa*, respectively (see Fig. 2a). Whereas, in penta-PdSe_2_ (see Fig. 2b) it is observed at 17$\mathrm{\%}$ (19$\mathrm{\%}$) with stress value of 3.46 *GPa* (3.40) *GPa*. The present values of UTSR for penta-PdQ_2_ are higher than the blue-phosphorene monolayer (~ 16$\mathrm{\%}$)^51^. Thus, the UTSR values suggest that the penta-PdQ_2_ monolayer is relatively more flexible.

### Electronic properties

**Figure 3:**
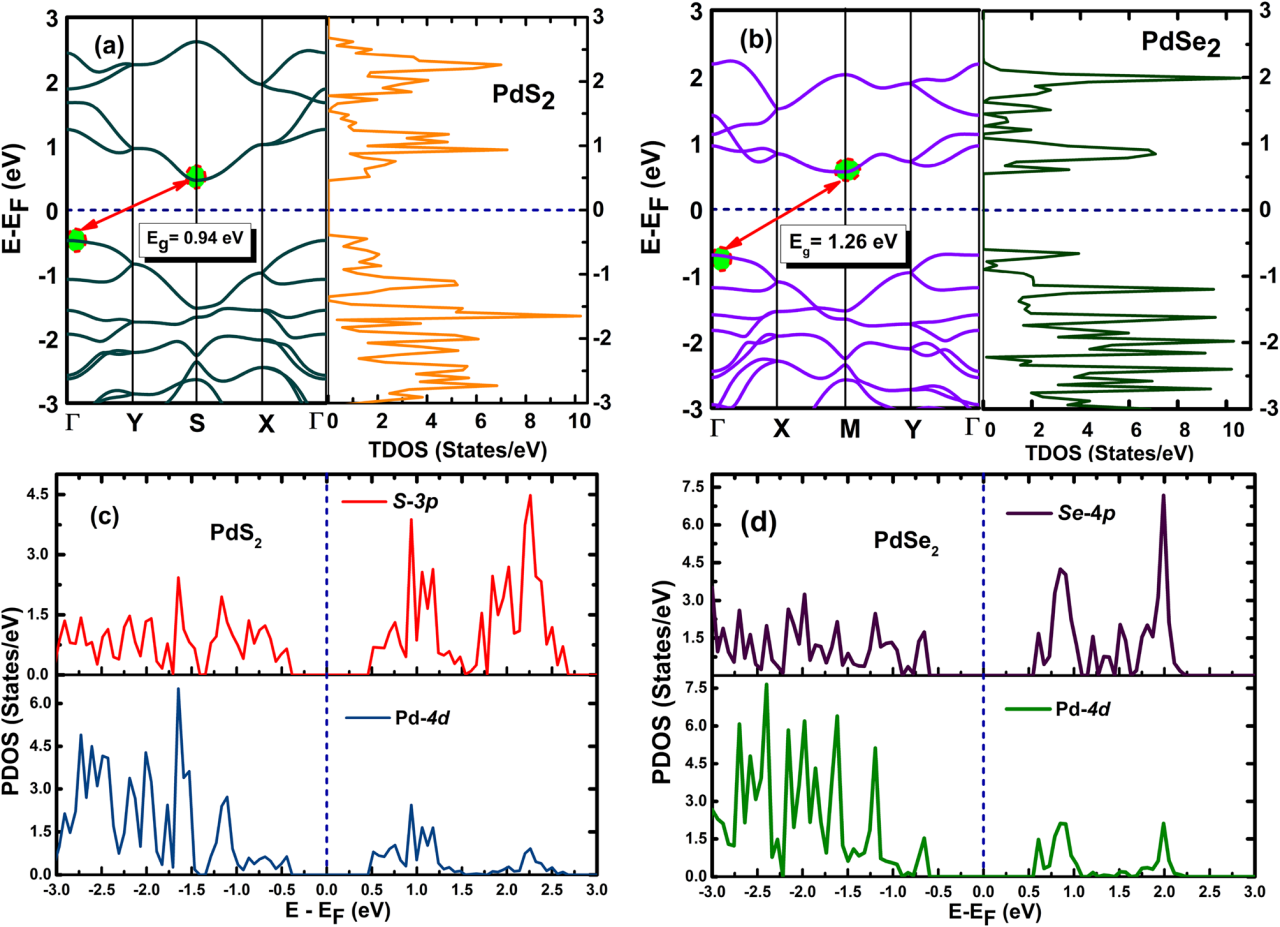
Electronic band structure and total density of states (TDOS) *States/eV* (**a**) For penta-PdS_2_ monolayer and (**b**) For penta-PdSe_2_ monolayer. The partial density of states (PDOS) *States/eV* (**c**) For penta-PdS_2_ monolayer and (**d**) For penta-PdSe_2_ monolayer.

The band structure, total density of states (TDOS) and partial density of states (PDOS) of penta-PdQ_2_ monolayers along high symmetry points of Brillouin zone (BZ) have also investigated. At PBE level, from Fig. 3a,b we can see that the penta-PdS_2_ (PdSe_2_) is an indirect band gap with valence band maximum (VBM) and conduction band minimum (CBM) tracing at S- point (M-point) and Γ-point, respectively. The obtained indirect band gap of penta-PdS_2_ is E_g_^PBE^ = 0.94 eV, while band gap for penta-PdSe_2_ is E_g_^PBE^ = 1.26 eV as seen in Fig. 3a,b, both the values are consistent with previously reported results^30,31^. As DFT-PBE functional may underestimate the bandgaps of semiconductors, we also used GGA + U, LDA, LDA + U and HSE06^52^ approximations to ensure calculation reliability and the obtained indirect electronic bandgap of PdS_2_ (PdSe_2_) are 1.05 (1.26) eV, 0.92 (1.31) eV, 0.94 (1.30) and 0.92 eV, respectively. For the present computations, the Hubbard parameter value taken as 3.69 eV^53^. Next, having heavier element (Pd) in 2D penta-PdQ_2_ material, the spin–orbit coupling (SOC) effect may play a crucial role and could influence in the band gap (*E*_*g*_) of material. Thus, we have also checked the SOC effect on the penta-PdQ_2_ monolayer and results are shown in Figure S4a,b, ESI^†^. Using PBE + SOC functional calculation, the obtained indirect band gap of penta-PdS_2_ is E_g_^PBE+SOC^ = 1.16 eV, whereas band gap of penta-PdSe_2_ is E_g_^PBE+SOC^ = 1.51 eV. Recently, the Yang et al.^32^, have reported electronic properties of penta-PdS_2_ from PBE (PBE + SOC) calculation and reported indirect band gap of 1.08 eV (1.13 eV), which is comparable with our results within of 12.96$\mathrm{\%}$ (11.5$\mathrm{\%}$) deviation. Moreover, a research group of Sun et al.^34^, have reported a band gap of penta-PdSe_2_ with PBE level theory and mentioned indirect band gap of 1.38 eV, which is higher by 8.6$\mathrm{\%}$ than our obtained band gap (~ 1.26 eV). Furthermore, with SOC effect, Qin et al.^33^, has computed indirect band gap of penta-PdSe_2_ monolayer via TB-mBJ-GGA potential and reported band gap 1.38 eV. The deviation of present band gap of penta-PdSe_2_ with PBE + SOC calculation is 9$\mathrm{\%}$. Further, the VBM and CBM are substantially coincide with each other regardless of PBE or HSE06 (with or without SOC) level calculations. Also, the HSE06 level calculations are really time expensive and need heavy computing power, as a results of that, we only used PBE functional level band structures for further calculations by applying strain. The nature of CBM and VBM are vary with applying strain on the PdQ_2_ monolayer to reach up the optimal value of 1.3 eV for solar cell applications, which is discussed in SQ Efficiency section. As presented in Fig. 3a,b, it is noticed that the TDOS (*States/eV*) are mostly ascend due to *‘d’* orbitals of Pd atom and *‘p’* orbitals of Q (S, Se) atom in the penta-PdQ_2_ monolayer, which are also consistent to the past reported results^32,34^. Additionally, the TDOS difference from PBE to PBE + SOC calculations are shown in Figure S5a,b, (ESI^†^) for PdS_2_ and PdSe_2_ monolayers. we have also investigated PDOS (*States/eV*) as displayed in Fig. 3 (c, d), to further investigate partial contribution of atoms in penta-PdQ_2_ and we conclude that the conduction bands (CBs) and valence bands (VBs) are highly occupied by the *4d-Pd* states and *3p-Q* (Q = S, Se) states, respectively.

### Transport properties

#### Carrier mobility

**Figure 4:**
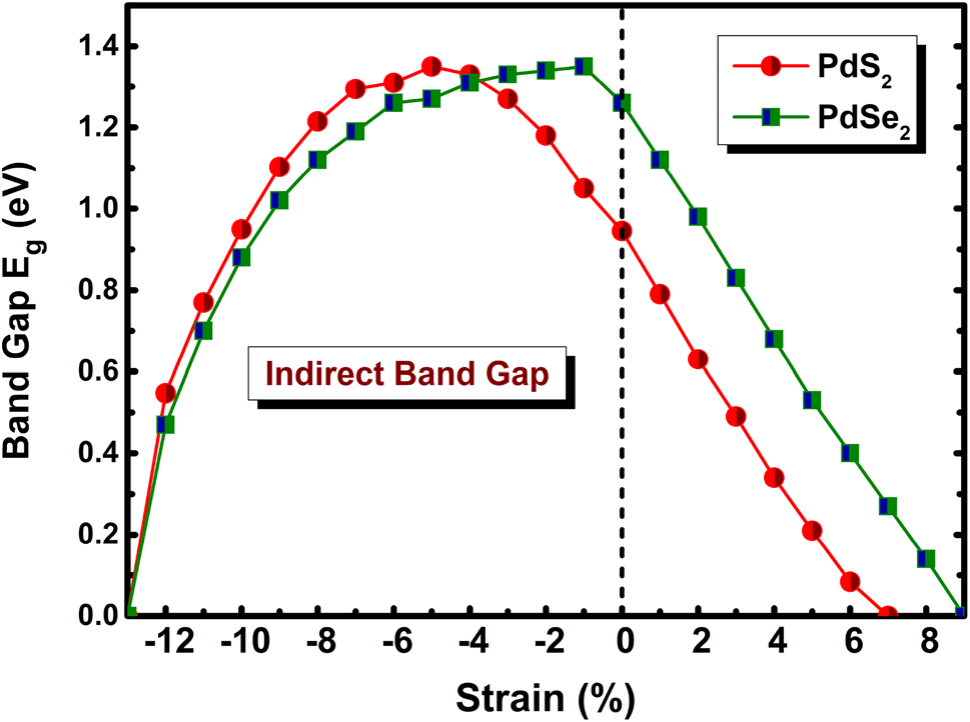
The variation of band gap E_g_ (*eV*) with applied biaxial strain $\upvarepsilon$ ($\mathrm{\%}$) on penta-PdQ_2_ monolayers.

We further focused on the band alignment in terms of strain effect to drive the electronic properties. Simultaneously, we have also calculated carrier mobility (*µ*) of monolayer, which is one of essential factor for a high-performance device like FET. For this, we have exerted compressive (-$\mathrm{\varepsilon \%}$) and tensile (+ $\mathrm{\varepsilon \%}$) strain on penta-PdQ_2_ (Q = S, Se) monolayers and traced influence on the band edges of CBM and VBM in the band structure, where system is anisotropic (*a* not equal to *b*). The strength of strain along biaxial direction is given by^54^, $\upvarepsilon$ = [(*S*−*S*_*0*_*)/S*_*0*_]$\times$100$\mathrm{\%}$; here, *S*_*0*_ is the unstrained lattice constant and *S* is the strained lattice constant of the monolayer. The response of applied strain to band gaps of penta-PdQ_2_ is shown in Fig. 4.

As stated before, the fundamental indirect band gap of penta-PdS_2_ and penta-PdSe_2_ is 0.96 eV and 1.26 eV, respectively. The Fig. 4 reflects that the band gap of penta-PdQ_2_ is gradually decreases almost linearly in presence of tensile strain (+ $\mathrm{\varepsilon \%}$) compared to fundamental band gap (0$\mathrm{\%}$). Interestingly, upon increases compressive strain (−$\mathrm{\varepsilon \%}$) band gap of penta-PdS_2_ increases up to -5$\mathrm{\%}$ compressive strain and then decreases gradually with respect to 0$\mathrm{\%}$. It is noteworthy that, the maximum value of band gap of penta-PdS_2_ is ~ 1.35 eV at $\upvarepsilon$ = −5$\mathrm{\%}$ and for penta-PdSe_2_ is ~ 1.36 eV at $\upvarepsilon$ = −1 $\mathrm{\%}$. Remarkably, the resistive phase transition is seen at higher compressive (−$\mathrm{\varepsilon \%}$) and tensile strain (+ $\mathrm{\varepsilon \%}$) in both cases. The semiconducting nature of penta-PdS_2_ (PdSe_2_) is transform into semi-metallic (~ 0 eV) by applying the biaxial compressive and tensile strain of −13$\mathrm{\%}$ (−13$\mathrm{\%}$) and + 7$\mathrm{\%}$ (+ 9$\mathrm{\%}$), respectively. Under the critical compressive strain (−$\mathrm{\varepsilon \%}$), the resistive phase change (semiconductor to semi-metallic) in penta-PdQ_2_ behaviour arises due to crossing one band of VBM at the Fermi level (Fig. S6a,b), ESI^†^). In contrary, up on the critical tensile (+ $\mathrm{\varepsilon \%}$) strain, it occurred due to touching one band of CBM at the Fermi level (Fig. S7a,b, ESI^†^). Additionally, we have also examined the phonon spectrum for each applied strain on penta-PdQ_2_ and computed its phonon frequency as summarized in Table S1, ESI^†^. Thus, our outcomes accomplish those electronic properties of studied 2D penta-PdQ_2_ (Q = S, Se) materials are tuneable by applying biaxial strain, which establish that the penta-PdQ_2_ is a promising candidate for designing flexible nanodevices.

Owing to tunability of penta-PdQ_2_ monolayers, we inspired to calculate the carrier mobility of penta-PdQ_2_ monolayers. For that the Bardeen and Shockley formalism^39^ had been applied, we have calculated the effective mass (m*), deformation potential *E*_*1*_ (*eV*) and stiffness constant *C*_*2D*_ (*J m*^*-2*^) to check the flow of charge carrier along biaxial strain. The carrier mobility depends on the most influenced factor on the carrier effective mass m* of electron (e) and hole (h), that is directly derived from the electronic band structure as^55^,

4 $${\mathrm{m}}^{*} = {{\hbar }}^{2}\left\{\frac{{\mathrm{d}}^{2}\mathrm{E}}{{\mathrm{dk}}^{2}}\right\}^{-1}$$

Next, deformation potential *E*_*1*_ is another vital parameter to effect carrier mobility and is obtained from the deformation theory (DP)^39^, which has been also successfully used method in previous reports for 2D MoS_2_, WS_2_ and phosphorene. The needed ingredients for carrier mobility (*µ*) are summarized in Table 2. Along biaxial strain, the obtained effective mass (m*) of electron and hole for penta-PdS_2_ (PdSe_2_) are 0.39 *m*_*e*_ (0.58 m_e_) and 0.72 *m*_*e*_ (0.14 *m*_*e*_), respectively. Our computed deformation potential *E*_*1*_ (eV) for penta-PdS_2_ is −6.44 eV for e and 6.84 eV for h, that is consistent with ones reported by Wang et al.^17^, along *x* or *y* direction. While in case of penta-PdSe_2_, the value of *E*_*1*_ is −2.91 eV for *e* and 6.14 eV for *h* that is also in accordance with the results of Qin et al.,^33^ for *x* or *y* direction. Whereas the in-plane stiffness constant *C*_*2D*_ for penta-PdQ_2_ has anisotropic behaviour. The in-plane stiffness constant obtained for penta-PdS_2_ and penta-PdSe_2_ are 121.76 J* m*^*-2*^ and 94.62 J* m*^*-2*^, respectively. The stiffness constant *C*_*2D*_ of penta-PdS_2_ is larger than the penta-PdSe_2_, indicating that PdSe_2_ monolayer is softer than the penta-PdS_2_ monolayer. As shown in Table 2, the achieved electron mobility of penta-PdS_2_ monolayer is 258 cm^2^ V^−1^ s^−1^, which is quite higher than the hole mobility ~ 67 cm^2^ V^−1^ s^−1^. Also, the carrier mobility ratio R comes out to be 0.26. Moreover, Wang et al.^17^ has reported the electron mobilities (cm^2^ V^−1^ s^−1^) of penta-PdS_2_ as 40.97 (*x*) and 169.11 (*y*), while hole mobilities (cm^2^ V^−1^ s^−1^) as 339.25 (*x*) and 91.73 (*y*). On other hand, if we compare carrier transport along biaxial strain of penta-PdSe_2_ the hole mobility is 1518 cm^2^ V^−1^ s^−1^ that is greater than the electron mobility ~ 442.49 cm^2^ V^−1^ s^−1^ and R is 3.43. However, it is observed that the hole mobility of penta-PdSe_2_ is higher than the 2D phosphorene^56^ (i.e., 640–700 cm^2^ V^−1^ s^−1^) and BN nanosheet (i.e., 500 cm^2^ V^−1^ s^−1^)^57^, indicating that penta-PdSe_2_ would be a promising material for modelling electronic applications.

**Table 2:** The carrier effective mass |m*| (m_e_, the mass of free electrons), deformation potential constant *E*_1_ (eV), stiffness constant *C*_*2D*_ (J m^−2^), carrier mobility (μ) for electron (e) and hole (h) and carrier mobility ratio *(R*) along biaxial direction of penta-PdQ_2_ monolayers at 300 K.

System	Carrier type	m*/m_e_	E_1_ (eV)	C_2D_ (J m^−2^)	μ (cm^2^ v^−1^ s^−1^)	R = μ_h_/μ_e_
Penta-PdS_2_	electron	0.39	−6.44	121.76	258.06	0.26
	hole	0.72	6.85	121.76	67.43	
Penta-PdSe_2_	electron	0.58	−2.91	94.62	442.49	3.43
	hole	0.14	6.14	94.62	1518	

#### Current–voltage (I-V) characteristics

**Figure 5:**
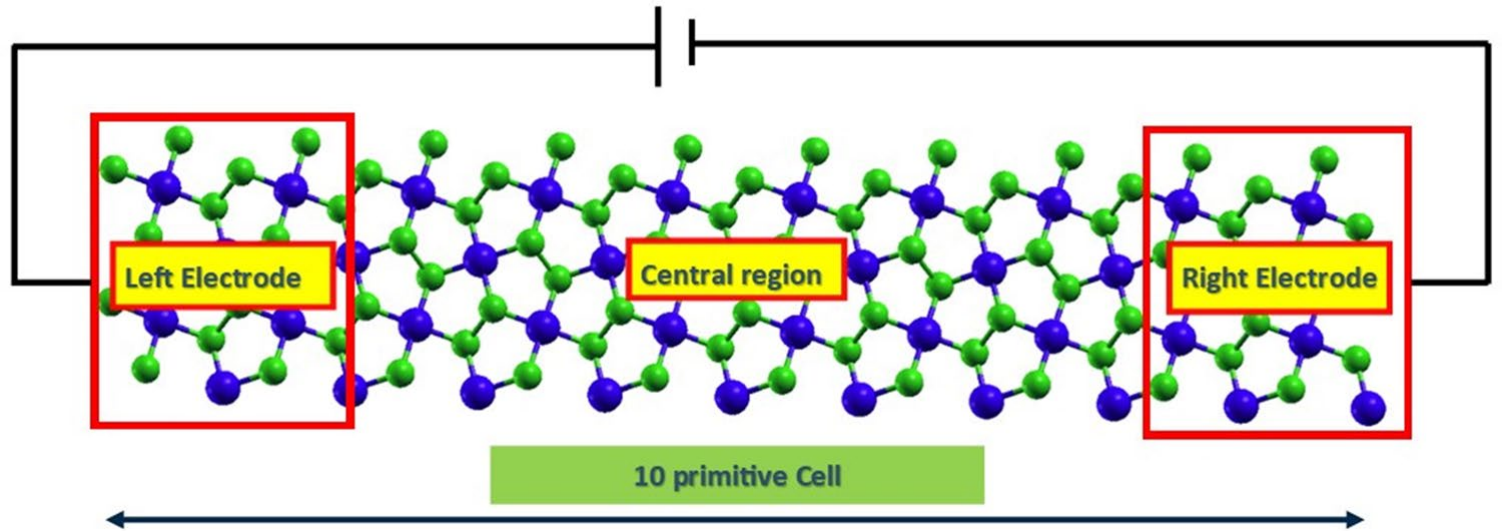
Schematic view of two-terminal device of penta-PdQ_2_ monolayer. Here electrons are driven from the cold to the hot lead through central region.

To understand the current sensitivity of the penta-PdQ_2_ monolayers, we have examined the I-V characteristics, based on the equivalent transport theory^41^. Figure 5 represents the schematic view of two-terminal device, where we have considered LE (left electrode), RE (right electrode) and scattering region of same material.

**Figure 6:**
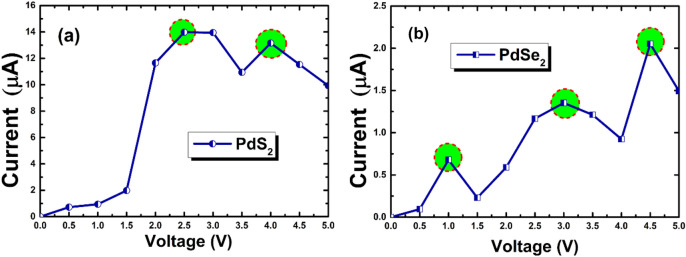
Current–voltage (*I-V*) plot of penta-PdQ_2_ under bias voltages from 0.0 V to 5.0 V. The negative differential conductance (NDC) regions are indicated by the highlighted circles.

Here, we have taken 10 primitive cells, among them the lead had built up with four-unit cell (24 atoms) and central region consist of 12-unit cells (72 atoms). The I-V characteristic of both penta-PdS_2_ and penta-PdSe_2_ monolayers are shown in Fig. 6a,b. It is seen that, no significant current is observed till ~ 1.0 V and 1.5 V then it starts to increase with increasing applied voltages due to semiconducting nature of penta-PdS_2_ and PdSe_2_ monolayers, respectively. Apparently, it can be seen form Fig. 6a,b penta-PdS_2_ is more current-sensitive than the penta-PdSe_2_ monolayer. Although, in the case of penta-PdS_2_, the first peak in current is seen at 2.5 V with 14 *µA* and second current peak is located at 4.0 V with current of 13.2 *µA*. This is indicating a pronounced negative differential conductance effect (NDC)^58^ in the bias range 2.0–3.5 V (see Fig. 6a). While in case of penta-PdSe_2_, the NDC effect is seen in range of 1.0–4.5 V (see Fig. 6b). More precisely, the first NDC effect is occurs at the bias voltage 1.0 V and current to be found about 0.678 µA. Further, the second and third peaks are occurring at the bias voltage of 3.0 V and 4.5 V with current up to 1.35 µA and 2.05 µA, respectively. The NDC effect is very useful feature in *I-V* curve because it would be playing a crucial role in the application of multipliers, mixers, logic gates, high-frequency oscillators, and A to D (analog-to-digital) converters^59^.

### SQ efficiency

**Figure 7:**
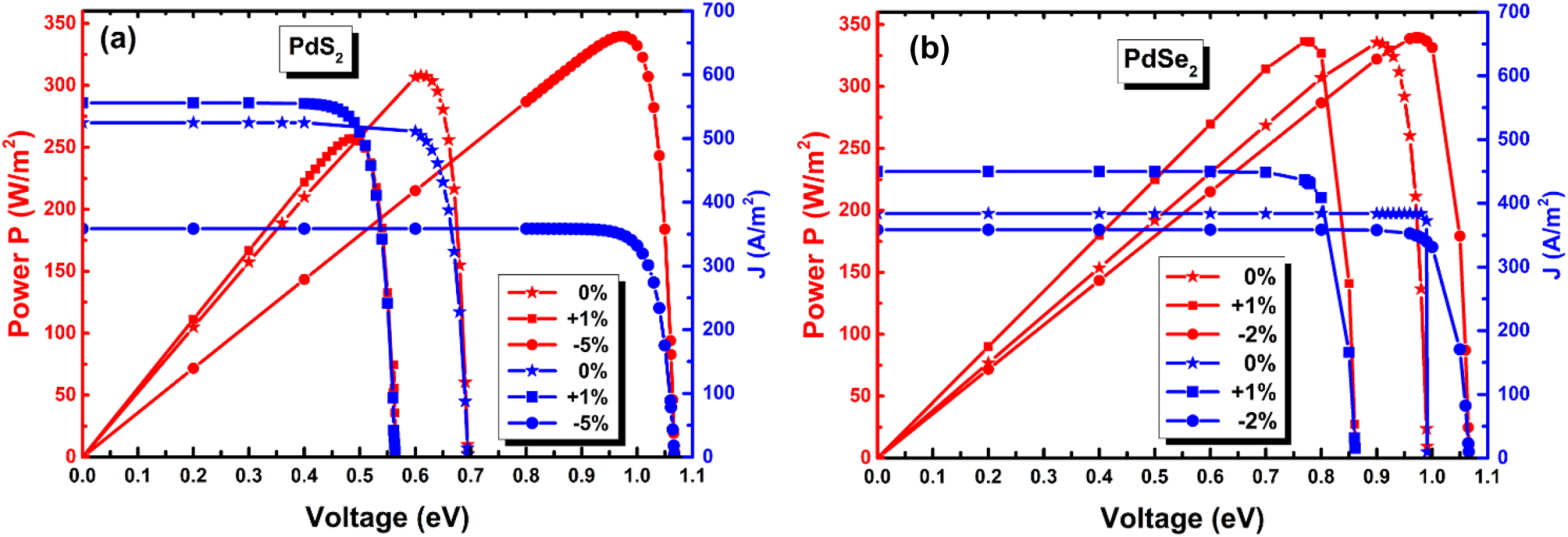
The calculated $J - V$ characteristic and power density curves of (**a**) for penta- PdS_2_ and (**b**) for penta-PdSe_2_ for pristine and compressive (-$\varepsilon \%$) or tensile (+ $\varepsilon \%$)) loaded strain (maximum efficiency $\eta$ ($\varepsilon \%$) of strain taken) at T = 300 K. _._

We also focused on the power conversion efficiency (PCE) of penta-PdQ_2_ (Q = S, Se) using Shokley-Queisser (SQ) limit that gives the percentage of power converted to electrical energy^60,61^. For this we required several parameters such as short circuit current density ($J_{sc}$), open circuit voltage ($V_{oc}$), maximum power density ($P_{max}$) and efficiency $\mathrm{\eta (\%)}$. The theoretical short circuit current density $J_{sc}$ is calculated using following relation^62,63^,

5 $$J_{sc} = \mathop \int \limits_{0}^{\infty } eA\left( E \right)I_{sun} \left( E \right)dE$$

where $e$ is the elementary charge, $A\left( E \right)$ represents the absorbance of layer^62^ and $I_{sun} \left( E \right)$ is the photon flux density as specified in the AM1.5G spectrum^64^, Also, the theoretical reverse saturation current $J_{0}$ is defined as $J_{0} = \mathop \int \limits_{0}^{\infty } e\pi A\left( E \right)I_{bb} \left( E \right)dE$^65^; Here $I_{bb} \left( {E,T} \right)$ is the black body spectrum and the radiative recombination fraction, which is supposed to be unity^60^. Thus, the total current density can be obtained using the formula^65^,

6 $$J = J_{sc} - J_{0} \left( {\exp \left( {\frac{e.V}{{k.T}}} \right) - 1} \right)$$

where $k$ is the Boltzmann’s constant, $V$ is the voltage over the absorber of the 2D devices. By the relation of $P = JV$, the maximum power density $P_{max}$ can be evaluated from the maxima of the $J - V$ curve, as presented in Fig. 7a,b. Eventually, with help of above-mentioned parameters, now we have obtained solar cell efficiency﻿ $\mathrm{\eta (\%)}$  by formula^59^,

7 $$\eta = \frac{{P_{max} }}{{P_{in} }}$$

where $P_{in} \left( {1000 \,{\text{W}}/{\text{m}}^{2} } \right)$ is total incident power density from solar irradiation of AM1.5G and $P_{max}$ is maximum power density.

**Figure 8:**
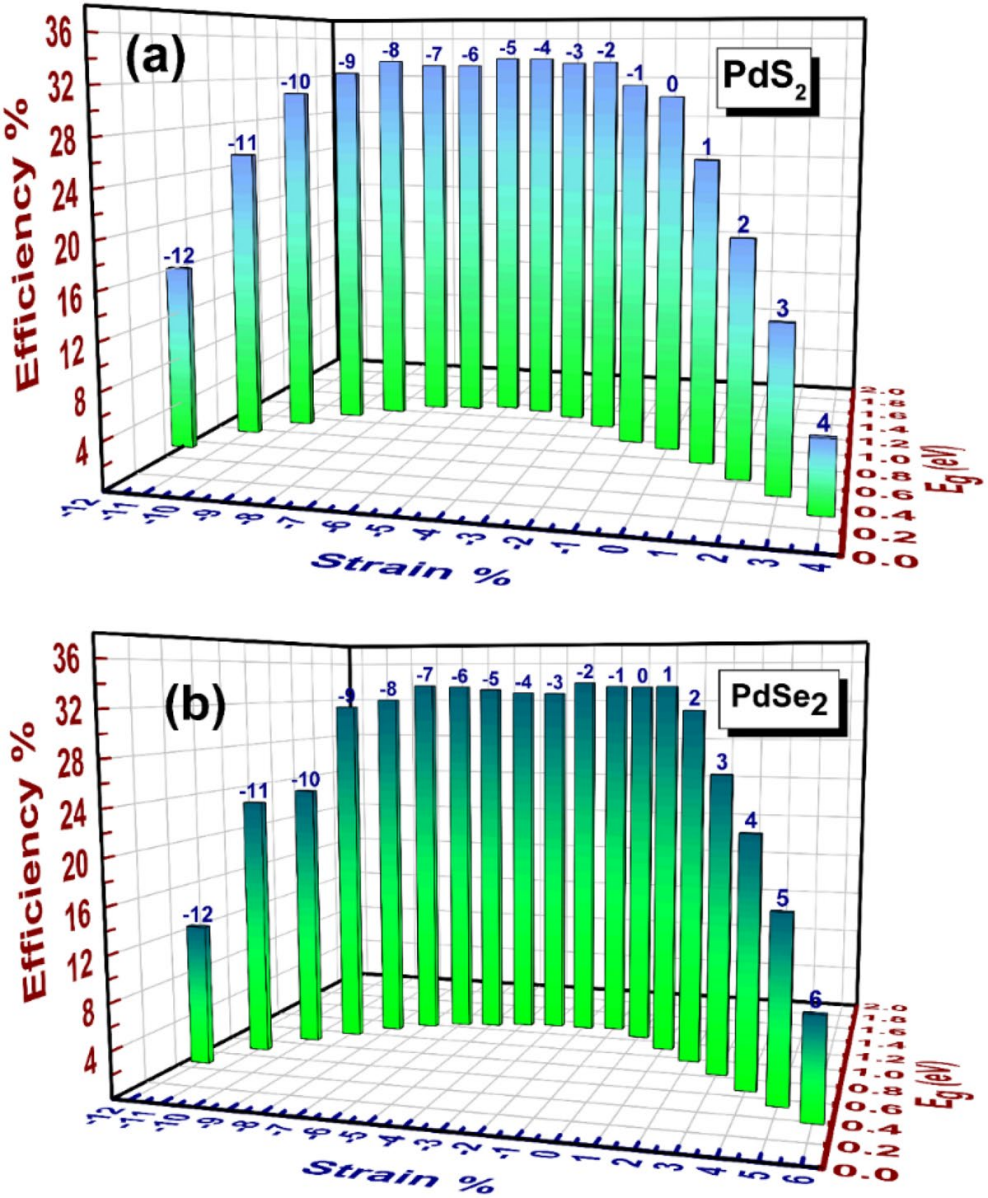
Change in SQ Efficiency η ($\%$) of penta-PdQ_2_ monolayers for pristine and loaded strain at T = 300 K (**a**) For penta-PdS_2_ and (**b**) For penta-PdSe_2_.

As mentioned in the SQ limit theory, the electronic band gap (*E*_*g*_) plays a pivotal role in the calculation of the material efficiency. Moreover, the maximum possible SQ efficiency limit is specified of 33.70$\%$ in circumstance of semiconductor with 1.34 eV optimum band gap^66^. As seen from the Table 3, the remarkable conversion efficiency for pristine penta-PdS_2_ and penta-PdSe_2_ is observed and are 30.79 and 33.54$\%$, respectively. Also, the efficiency of penta-PdSe_2_ is larger than the efficiency of benchmark GaAs (~ 31.4$\%$) solar cell with single junction. Next, the band gap of both monolayers could be well tuned from 0.34 eV ~ 1.35 eV under influence of biaxial strain ($\%$) as shown in Fig. 8a,b. Therefore, we have also checked the conversion efficiency under compressive (−$\varepsilon \%$) and tensile strain (+ $\varepsilon \%$) for both the monolayers and presented in Fig. 8a,b. It is noted that, when we have exerted compressive strain (−$\varepsilon \%$) on the penta-PdQ_2_ the efficiency at certain strain is higher as compared to the pristine (0 $\%$), that of 33.93 $\%$ (at −5 $\%$ of penta-PdS_2_) and −33.94 $\%$ (at −2 $\%$ of penta-PdSe_2_). On other hand, under tensile strain the maximum efficiency arises that of 25.72 $\%$ at + 1 $\%$ of penta-PdS_2_ and 33.63 $\%$ at + 1 $\%$ of penta-PdSe_2_, which is lower than with respective each of pristine (0 $\%$). This suggests that, by applying biaxial strain on the penta-PdQ_2_ the related SQ efficiency could be tuned. Also, the fabrications of penta-PdQ_2_ solar cell have potential to improve photovoltaic performance compared to traditionally use Si-based solar cells.

**Table 3:** The calculated values of $J_{sc}$*(A/m*^*2*^*)*, $P_{max}$ *(W/m*^*2*^*)* and $\mathrm {\eta (\%)}$ of penta-PdQ_2_ (Q = S, Se) for both pristine and loaded strain ($\%$). Significant values are in bold.

2D-System	Strain ($\%$)	${\varvec{J}}_{{{\varvec{sc}}}}$(A/m^2^)	${\varvec{P}}_{{{\varvec{max}}}}$(W/m^2^)	Efficiency $\user2{\eta (\%) }$
Penta-PdS_2_	0%	524.67	307.96	**30.79%**
	+ 1%	555.79	257.2	25.72%
	−5%	358.48	339.3	33.93%
Penta-PdSe_2_	0%	383.82	335.4	**33.54%**
	+ 1%	449.66	336.3	33.63%
	−2%	358.48	339.48	33.94%

### Optical properties

**Figure 9:**
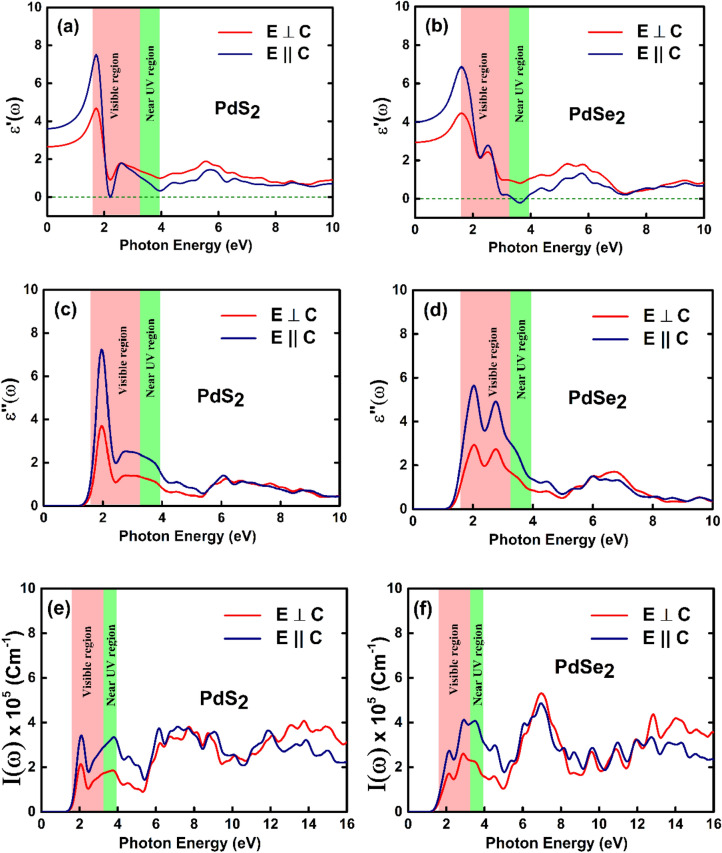
Computed optical properties of penta-PdQ_2_ monolayers for parallel and perpendicular polarization (**a**,**b**) real ($\varepsilon$’) and (**c**,**d**) imaginary ($\varepsilon$”) parts of complex dielectric function. (**e**,**f**) absorption coefficient ${\rm I}\left( \omega \right)$.

In order to evaluate light absorption ability of penta-PdQ_2_, we have calculated the real ($\varepsilon$’) and imaginary ($\varepsilon$”) dielectric functions, absorption coefficient ${\rm I}\left( \omega \right)$, refractive index $n\left( \omega \right)$, and reflectivity $R \left( \omega \right)$ of penta-PdQ_2_ monolayers with parallel (*E* $\parallel$ *c*) and perpendicular (*E* $\bot$ *c*) electric vector (E). The computation is carried out using the Kramers-Kroning (KK) relationship^67^ and results are shown in Figs. 9, S8 and S9, ESI†.

In present work, the computed dielectric constants are shown in the large range of photon energy (*ħω*) from 0 to 10 eV and absorption coefficients ${\rm I}\left( \omega \right)$ in 0 to 16 eV. The visible (*VIS*; 1.59 – 3.26 eV) and near *UV* (*UV-A*; 3.26—3.94 eV) spectrum regions are shaded by the light-pink and light-green colour, respectively. Figure 9a,b shows the graphical image of real ($\varepsilon$’) and imaginary ($\varepsilon$”) components of penta-PdQ_2_, that simply gives the idea about the electronic polarizability of the material from the Clausis—Mossotti ^68^ relation. It can be seen from the Fig. 9a,b, the static dielectric constant ($\varepsilon_{\parallel }$(0), $\varepsilon_{ \bot }$(0)) of penta-PdS_2_ is (3.56, 2.62), while static dielectric constant of penta-PdSe_2_ is 4.0 ($\varepsilon_{\parallel }$(0)) and 2.94 ($\varepsilon_{ \bot }$(0)). This means that its $\varepsilon$(0) of parallel optical vector have more dominant polarizability compared to the perpendicular of $\varepsilon$(0). The optical peculiarity is strongly connected to the imaginary part of the dielectric function $\varepsilon$”(ω), which reflects the transition between the energy bands near the *E*_*F*_ levels and that would be govern the linear response of the material to light under small wave vector as depicted in Fig. 9 (c, d). Therefore, the imaginary part $\varepsilon$”(ω) in case of penta-PdS_2_, the one major peak occurred at 1.98 eV in the visible (*VIS*) region for both $\varepsilon_{ \bot } \left( \omega \right)$ and $\varepsilon_{||} \left( \omega \right)$ case as plotted in Fig. 9c. While, in case of penta-PdSe_2_, it is clearly seen (Fig. 9d) that there are two intense peaks appeared in the visible region (*VIS*) at *ħω* = 2.04 eV and 2.76 eV due to the electronic transition of *‘4p’* states of Se atom and *‘4d’* states of Pd atom between conduction bands (CBs) and valence bands (VBs) of the electronic band structure. Apparently, the peaks tendency of $\varepsilon_{ \bot } \left( \omega \right)$ and $\varepsilon_{||} \left( \omega \right)$ are similar on the photon energy spectrum for both penta-PdQ_2_ monolayers.

The optical absorption spectra of the penta-PdQ_2_ were recorded by absorption coefficient ${\rm I}\left( \omega \right)$ vs photon energy (*eV*) as shown in Fig. 9e,f. For penta-PdQ_2_, the first absorption peak orientation is in the ~ 1.5–5 eV and second broad peak’s orientation is cover the *UV* region up to photon energy of 16 eV. For penta-PdS_2_, the low energy excitonic peak occur around 2.1 eV in infrared region (*IR*) and that peak rise towards the visible region at 3.78 eV for both the polarization. The maximum absorption coefficient ${\rm I}\left( \omega \right)$ of penta-PdS_2_ for parallel and perpendicular polarization is 3.65 × 10^5^ and 4.04 × 10^5^, respectively (See Fig. 9e). While, in case of penta-PdSe_2_, the first peak is located at 2.2 eV in the *IR* region and kick off in visible region at highest absorption coefficient ${\rm I}\left( \omega \right)$ of 4.12 $\times$ 10^5^. However, with perpendicular polarization the highest coefficient found is about 5.28 $\times$ 10^5^ in the *UV* region (See Fig. 9f). Thus, the penta-PdQ_2_ monolayers have shown prominent absorption in *VIS* and *UV* region, which suggest a good prospect of penta-PdQ_2_ monolayers as *UV* filters and *UV* photodetectors.

Next, the computed refractive index $n\left( \omega \right)$ and reflectance $R\left( \omega \right)$ of penta-PdQ_2_ have the same evolution curve as plotted in Figure S8 (a, b), ESI^†^ and S9 (a, b), ESI^†^. In penta-PdS_2_, we observed static refractive index as ($n^{\parallel }$(0), $n^{ \bot }$(0)) = (1.90, 1.63) (see Figure S8 (a), ESI^†^). With the parallel polarization, the $n\left( \omega \right)$ is rising in the VIS region up to *ħω* = 1.78 eV, then going down up to energy of 2.32 eV. Similarly, in case of perpendicular polarization it has also gone down in the *VIS* region with 1.23 of n (ω) by the same spectrum photon energy. Moreover, the reflectivity $R\left( \omega \right)$ is also shown in Figure S9 (a), ESI^†^. The static reflectance ($R_{\parallel } \left( 0 \right),R_{ \bot } \left( 0 \right)$) of penta-PdS_2_ in *VIS* region is about to (10$\%$, 5$\%$) and the most elevated peak of reflectance is found to be in the *VIS* region with reflectance of 31*%* as seen in Figure S9 (a, b), ESI^†^. While, for penta-PdSe_2,_ the static refractive index ($n^{\parallel }$(0), $n^{ \bot }$(0)) is (2.0, 1.71) as shown in Figure S8 (b), ESI^†^, which is also in good agreement with previous reported work on penta-PdSe_2_ monolayer by the Zhao et al.^69^ group. The first and highest peak of $n\left( \omega \right)$ is trapped in the VIS region at energy of 1.68 eV, at that energy the $n^{\parallel }$(ω) is 2.65. The intense peak of $n^{ \bot }$(ω) is also located at *ħω* = 1.68 eV in the *VIS* region with $n^{ \bot }$(ω) of 1.68. The static reflectance ($R_{\parallel } \left( 0 \right),R_{ \bot } \left( 0 \right)$) of penta-PdSe_2_ in *VIS* region is about to (11$\%$, 7$\%$) as seen in Figure S9 (b), ESI^†^. Also, the highest peak of $R_{ \bot } \left( \omega \right)$ is occurred in the *VIS* region with the reflectance of 26$\%$, that reflectance is in good agreement with Zhao et al.^69^ reported results of penta-PdSe_2_ monolayer. Overall, the prominent peaks appeared in the $n\left( \omega \right)$ and $R \left( \omega \right)$ in the *VIS* region, that means most of incident light energy is reflected and refracted in *VIS* region and only small amount of incident energy is absorbed by the material.

## Conclusions

In the summary of the work, the first principles method has been employed to study the band structure, bulk modulus, Young’s modulus, transport properties such as carrier mobility and *I-V* curve, and optical properties of penta-PdQ_2_ (Q = S, Se) monolayers. The positive phonon spectrum gives the ground state dynamical stability of both the materials. The obtained electronic band gap is 0.94 eV and 1.26 eV for penta-PdS_2_ and penta-PdSe_2_, respectively. From mechanical stability point of view, penta-PdS_2_ withstands up to 16$\%$ (18$\%$) in *x* (*y*) directions, while penta-PdSe_2_ is up to 17$\%$ (19$\%$), which undoubtedly implies that each monolayer is flexible up to that strain. Interestingly, the *I-V* curve shows the NDC effect beyond the bias voltage of 2.5 V (for PdS_2_) and 3.0 V (for PdSe_2_), hence this feature leads us to conclude that penta-PdQ_2_ monolayers will be consider in future as promising material for NDC-based nanodevices. More importantly, the SQ efficiencies for pristine PdS_2_ and PdSe_2_ were 30.79$\%$ and 33.54$\%$, respectively, that could be essential utilized in the solar cell application. The computed optical properties reveal that the absorption range of penta-PdQ_2_ is very broad in *UV* regions. The maximum absorption coefficient ${\rm I}\left( \omega \right)$ of penta-PdS_2_ and penta-PdSe_2_ are found in the *UV* region are 4.04 $\times$ 10^5^ and 5.28 $\times$ 10^5^, respectively. Collectively, due to unique electronic, mechanical, transport and optical properties of penta-PdQ_2_ monolayers, the 2D materials have application prospect in the arena of semiconducting nanodevices.

## Supplementary Information

Supplementary Information.
