# Supplementary material for: Strain modulating electronic band gaps and SQ efficiencies of semiconductor 2D PdQ2 (Q = S, Se) monolayer
Source: Sci Rep. 2022 Feb 22;12:2964. doi: 10.1038/s41598-022-06142-6 (PMC8863876; doi:10.1038/s41598-022-06142-6)
Supplement: Supplementary file 1 — Supplementary Information. [file 41598_2022_6142_MOESM1_ESM.docx]

# SUPPLEMENTARY MATERIAL

**Strain Modulating Electronic Band Gaps and SQ Efficiencies of Semiconductor 2D PdQ_2_ (Q= S, Se) monolayer**

Dhara Raval^1^, Sanjeev K. Gupta^2, *^, P. N. Gajjar^3,^ **^*^** and Rajeev Ahuja^4^

^1,3^Department of Physics, University School of Sciences, Gujarat University, Ahmedabad 380009, India.

^2^Computational Materials and Nanoscience Group, Department of Physics and Electronics, St. Xavier's College, Ahmedabad 380009, India.

^3^Ångströmlaboratoriet, Department of Physics and Astronomy, Uppsala University, Lägerhyddsvägen 1, Box 516, 751 20 Uppsala, Sweden.

*(4^th^ January, 2022)*

**Keyword (s):** 2D monolayer, Density functional theory, Band structure, Carrier mobility, SQ efficiency

^*^Corresponding authors: Prof. (Dr.) P. N. Gajjar (E-mail: pngajjar@gujaratuniversity.ac.in, pngajjar@rediffmail.com) and Dr. Sanjeev K. Gupta (E-mail: sanjeev.gupta@sxca.edu.in)

**
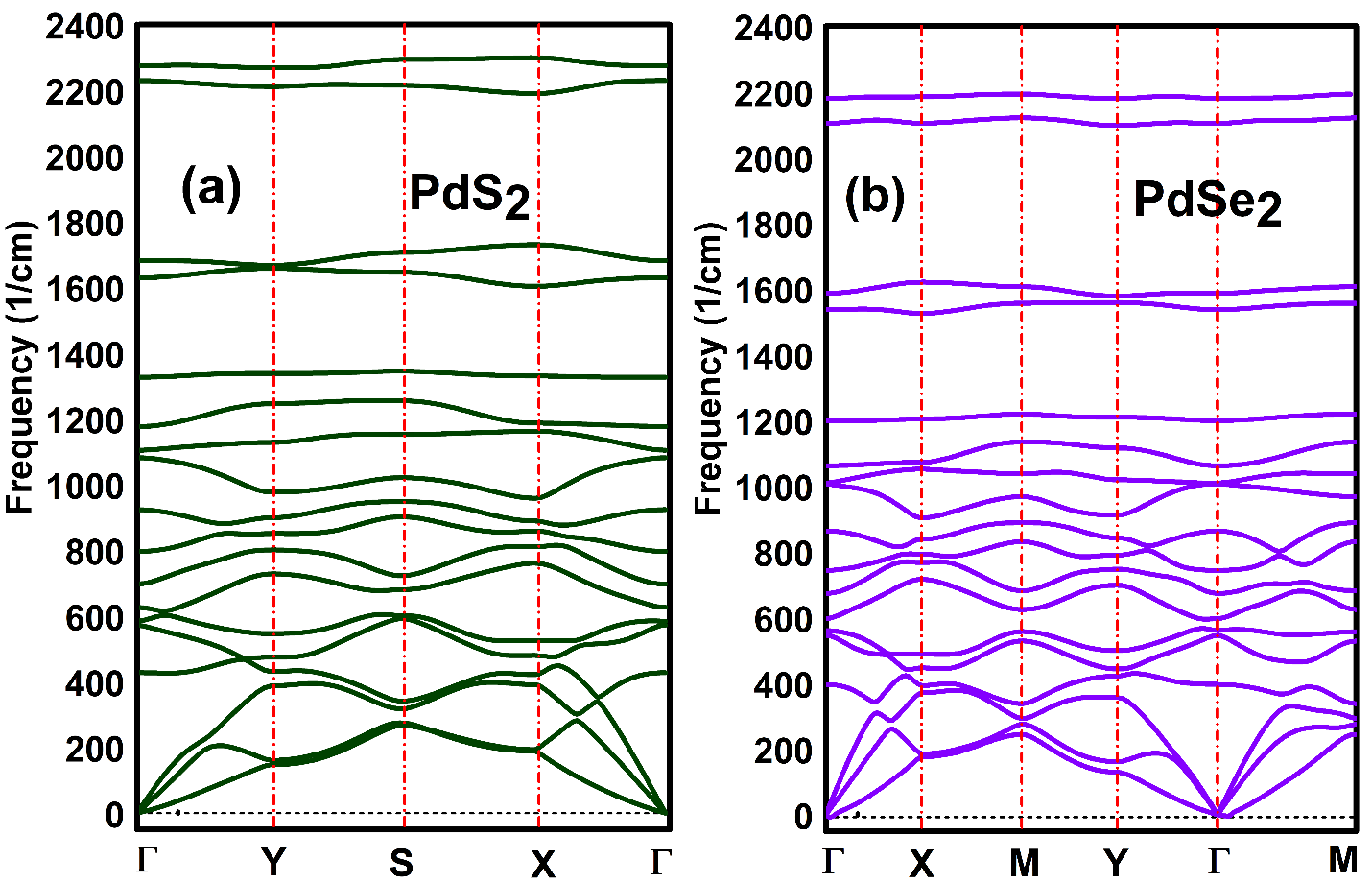
**

**Figure S1.** (colour online) Phonon spectrum of penta-PdQ_2_ monolayers. (a) For penta-PdS_2_ and (b) For penta-PdSe_2_.

Figure S1 shows the Phonon dispersive curves of penta-PdQ_2_ monolayer. Where system has six atoms threfore their phonon dispersion curves (longitudinal and transveres) consists with 18 modes of which 3 are acoustical modes (LA and TA) and 15 are optical modes (LO and TO). The extremely energatic phonon branches may be due to the phonon calculated within the consideration of dense k-grid points (20x20x1) and band lines.


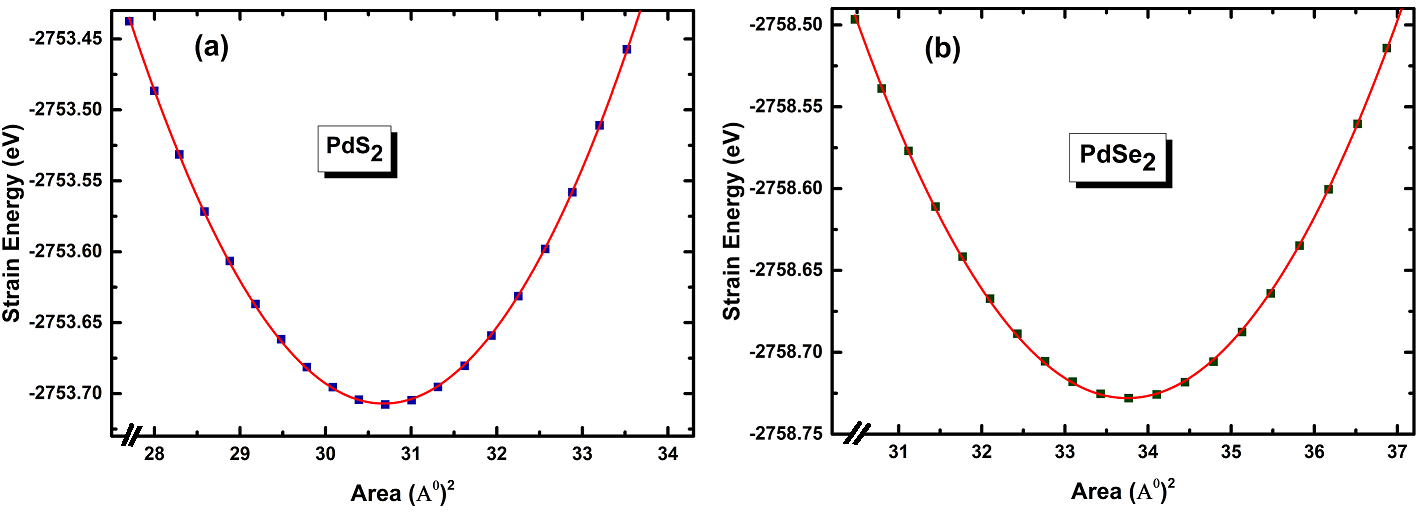


**Figure S2.** (colour online) The Strain energy (eV) vs area (Å^2^) curve under unstrained and loaded structures. (a) For penta-PdS_2_ and (b) For penta-PdSe_2_


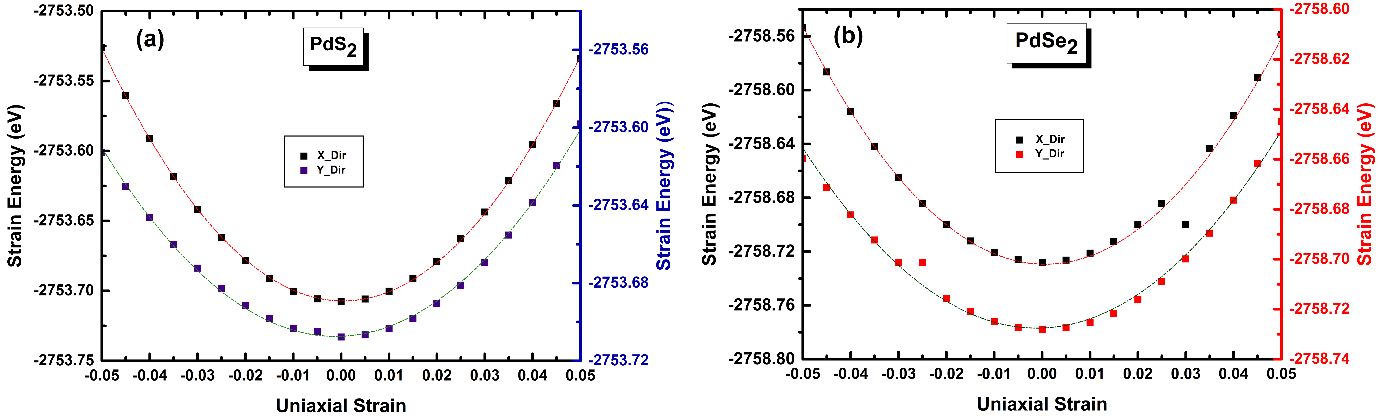


**Figure S3.** (colour online) The strain energy (eV) vs uniaxial strain (x or y) curve under pristine and loaded structures. (a) For penta-PdS_2_ and (b) For penta-PdSe_2_


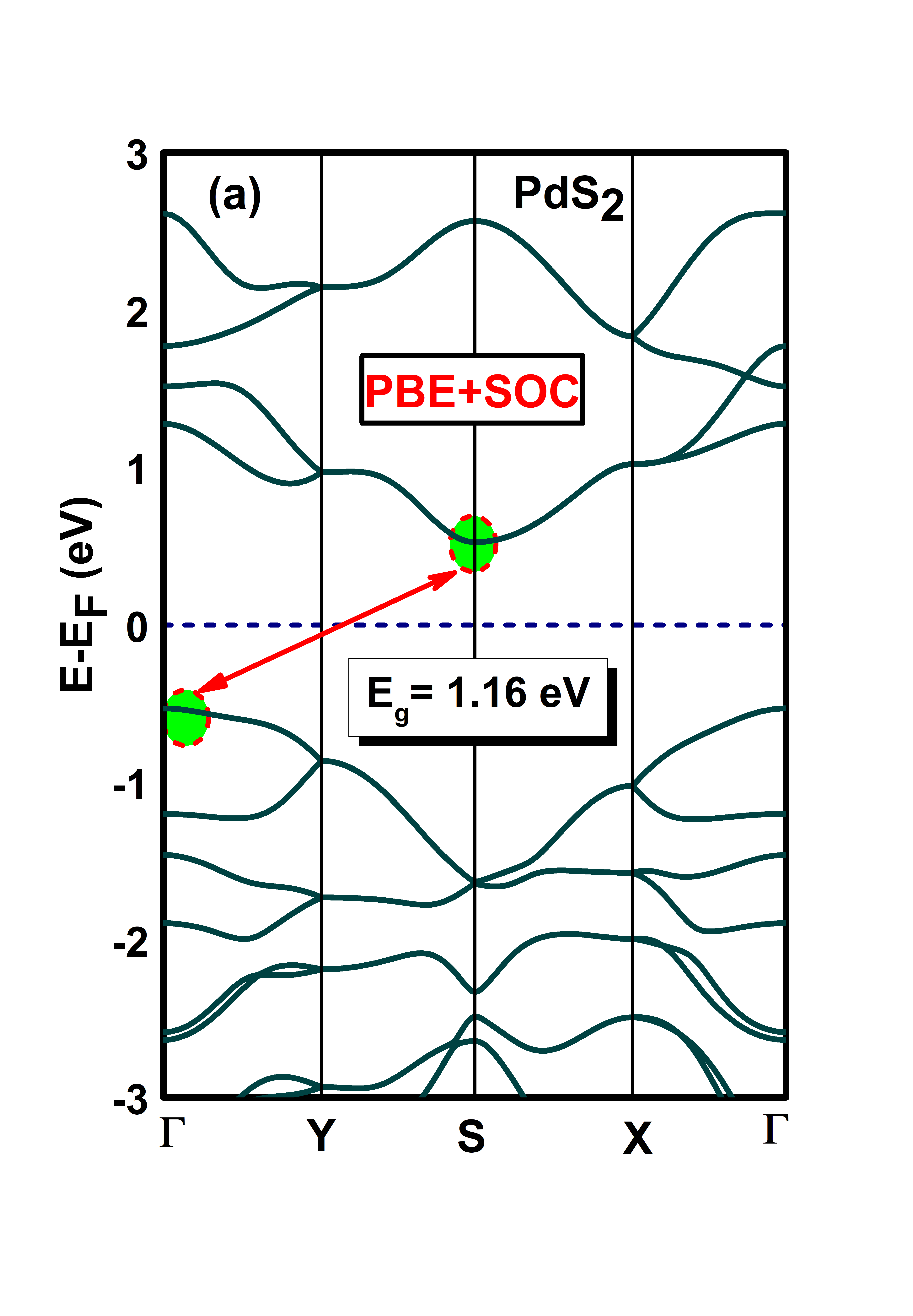

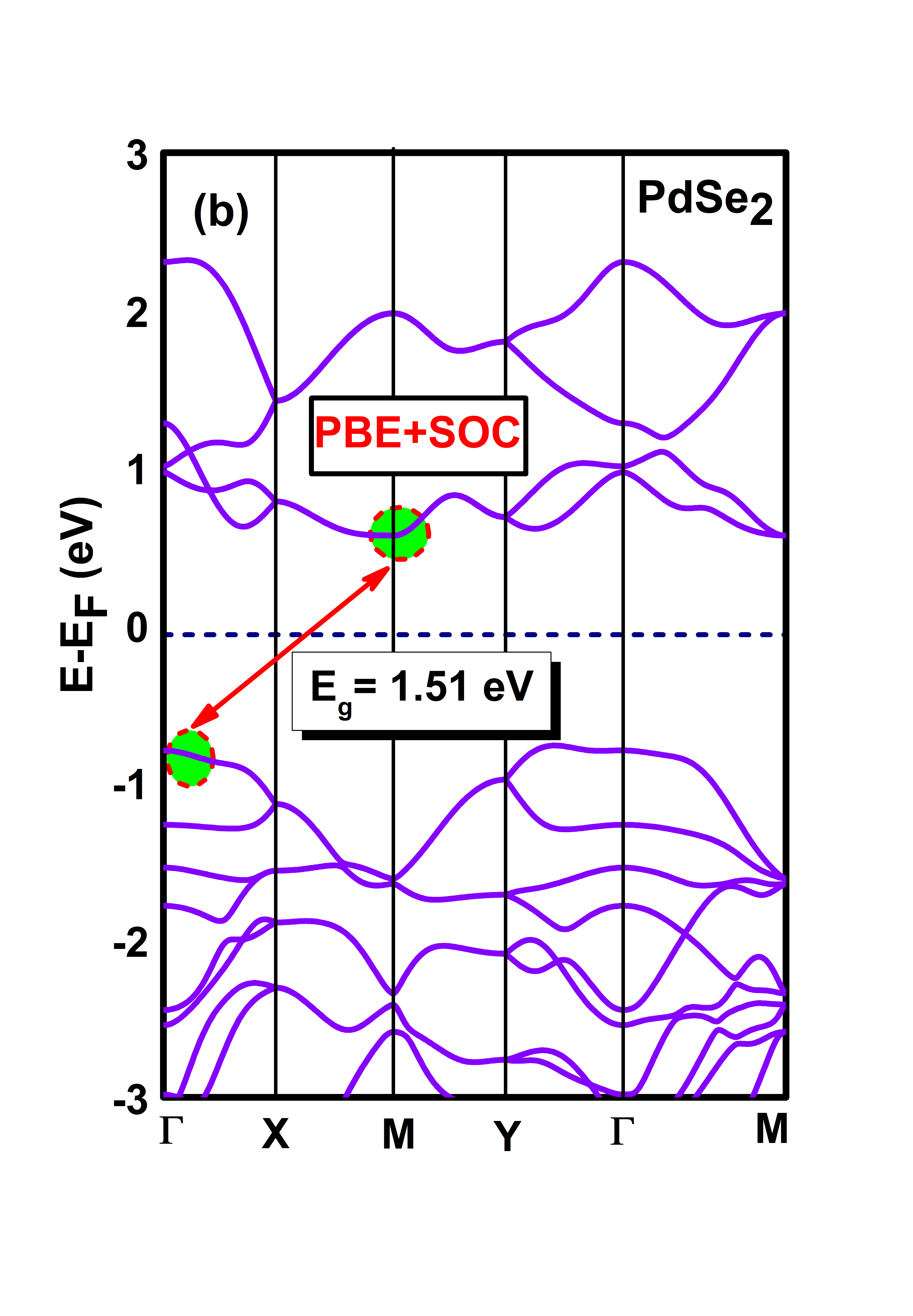


**Figure S4.** (Colour online) Electronic band structure of 2D-penta-PdQ_2_ with PBE+SOC. (a) For penta-PdS_2_ monolayer and (b) For penta-PdSe_2_ monolayer.


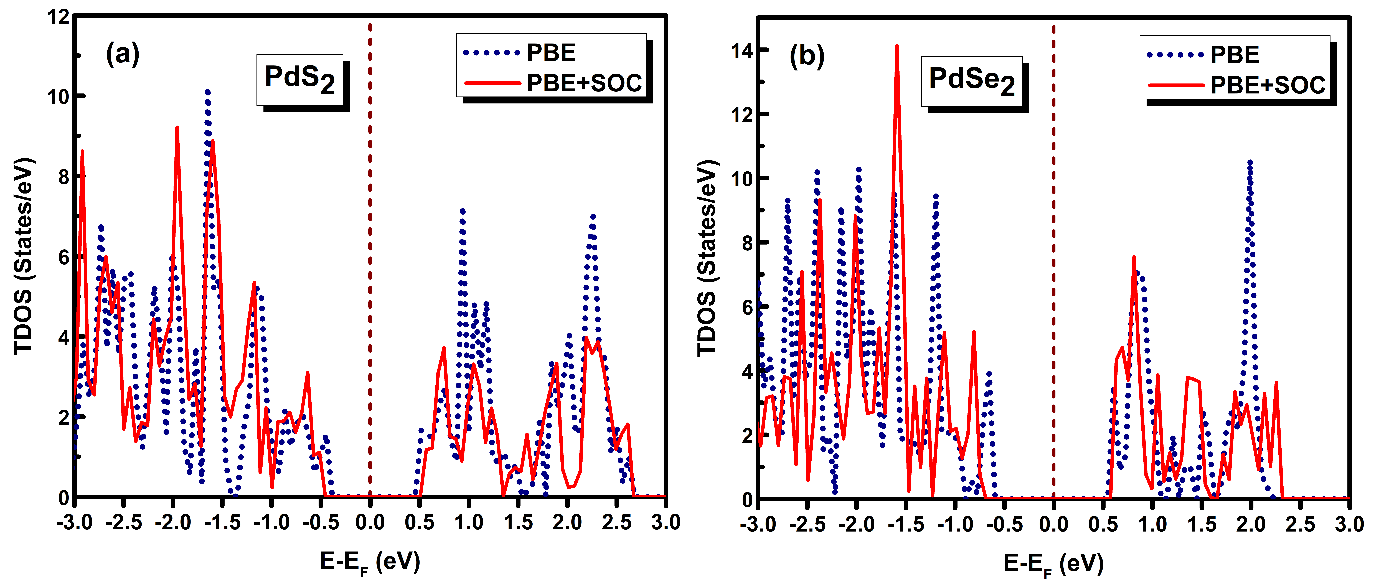


**Figure S5.** (Colour online) Total density of states (TDOS) States/eV with PBE and PBE+SOC calculations. (a) For penta-PdS_2_ monolayer and (b) For penta-PdSe_2_ monolayer.

| 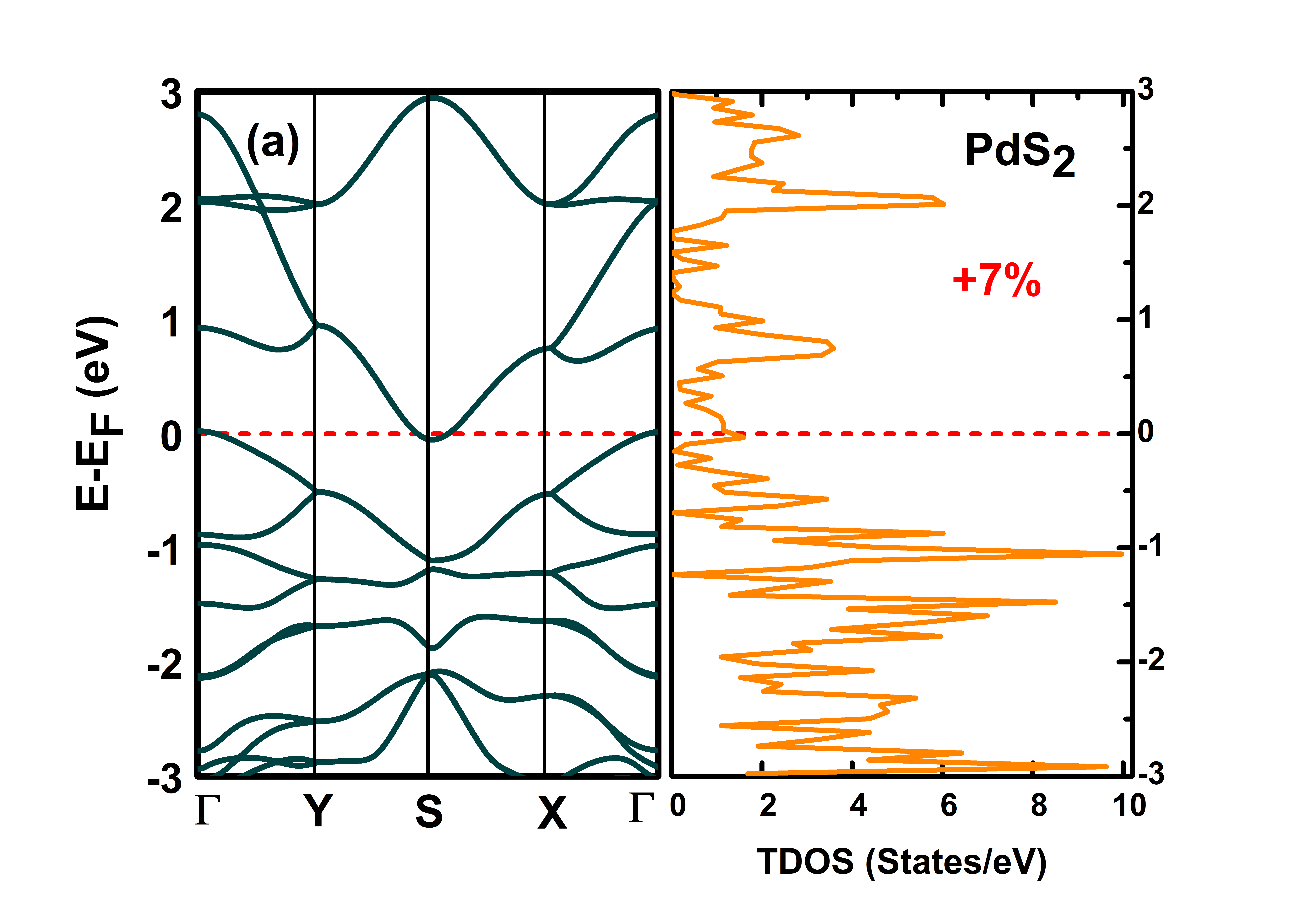 | 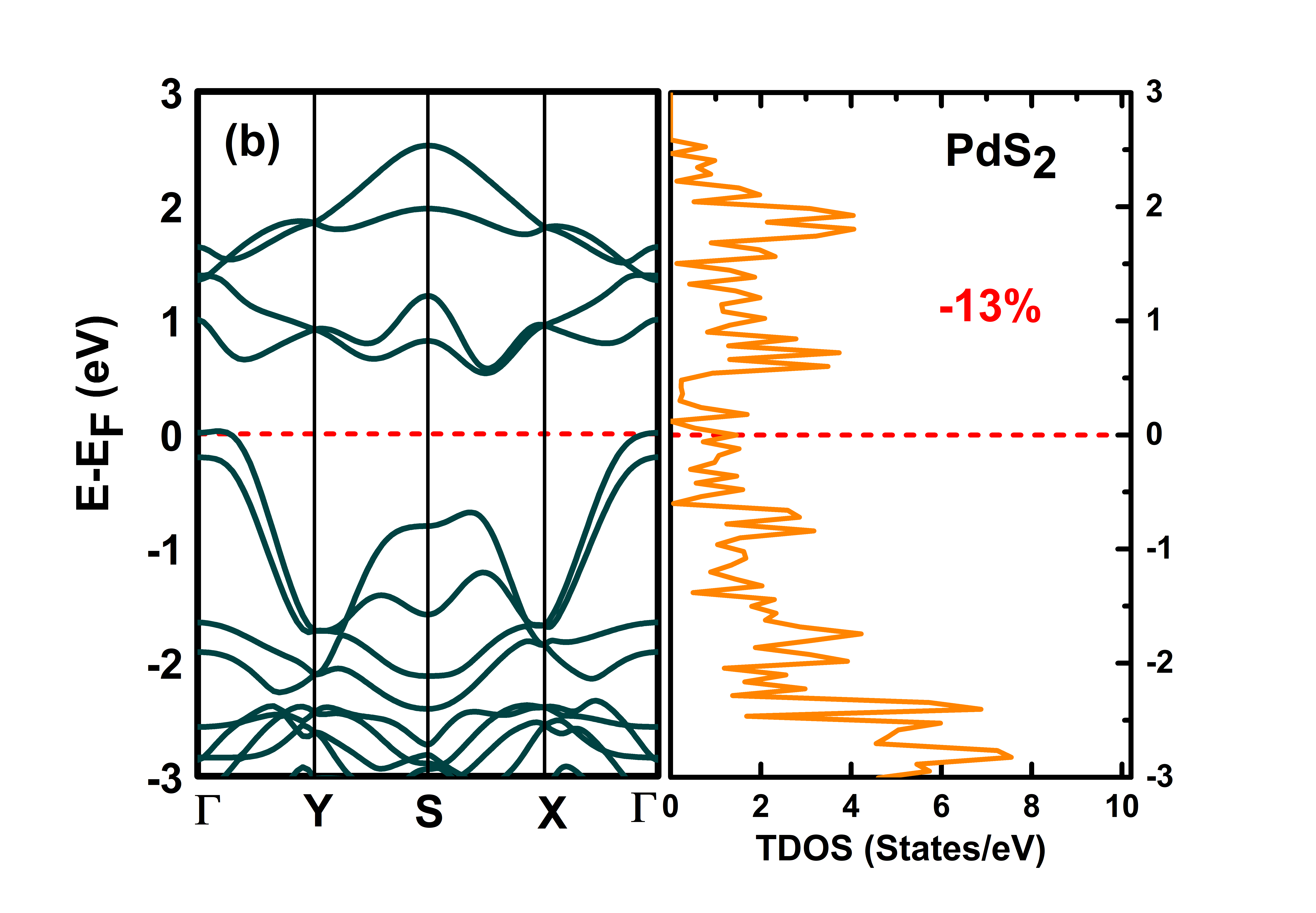 |
| --- | --- |

**Figure S6.** (Colour online) Electronic band structure and total density of states (TDOS) States/eV. (a) For penta-PdS_2_ monolayer under critical tensile strain (+7%) and (b) For penta-PdS_2_ monolayer under compressive strain (-13%).

| 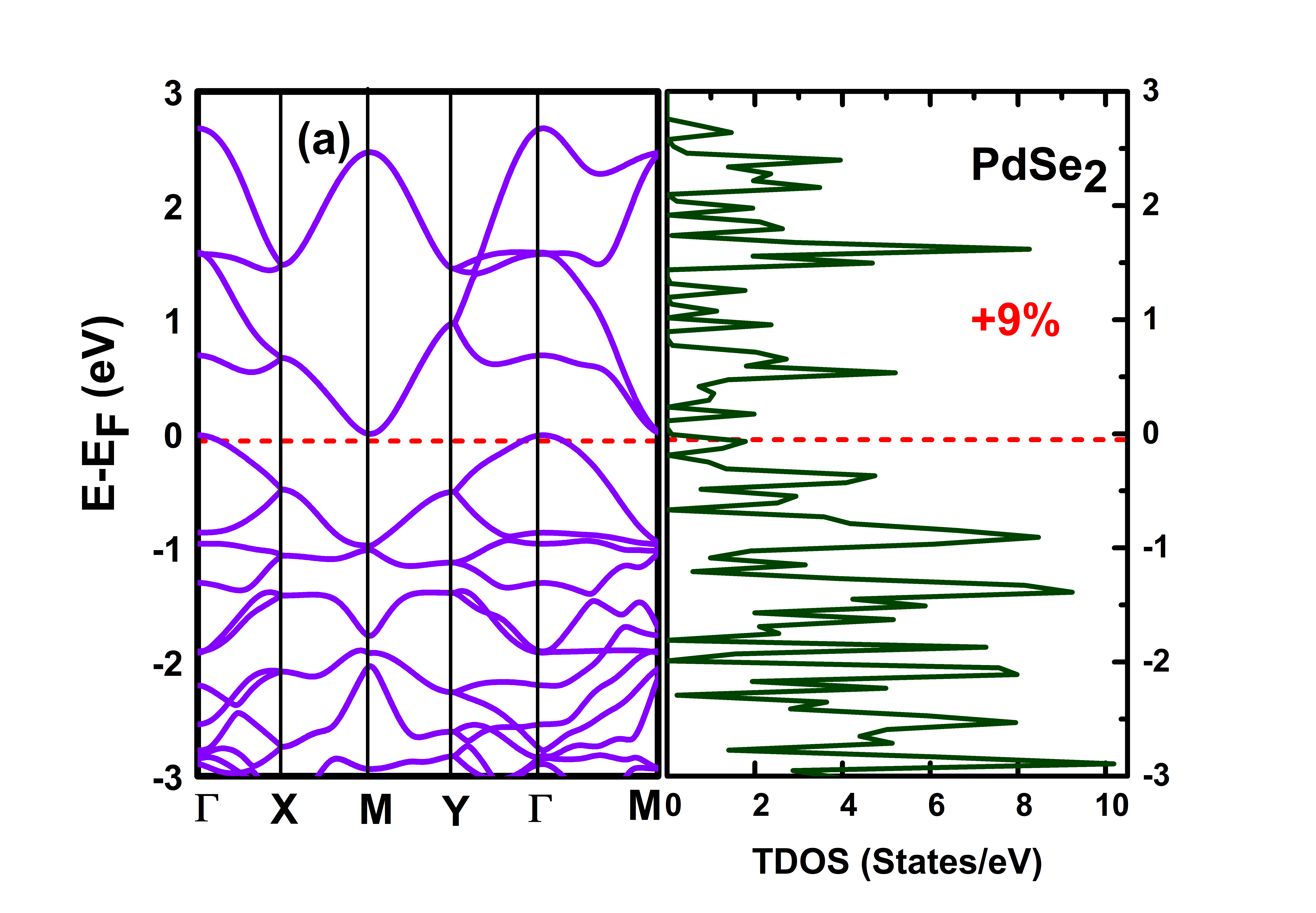 | 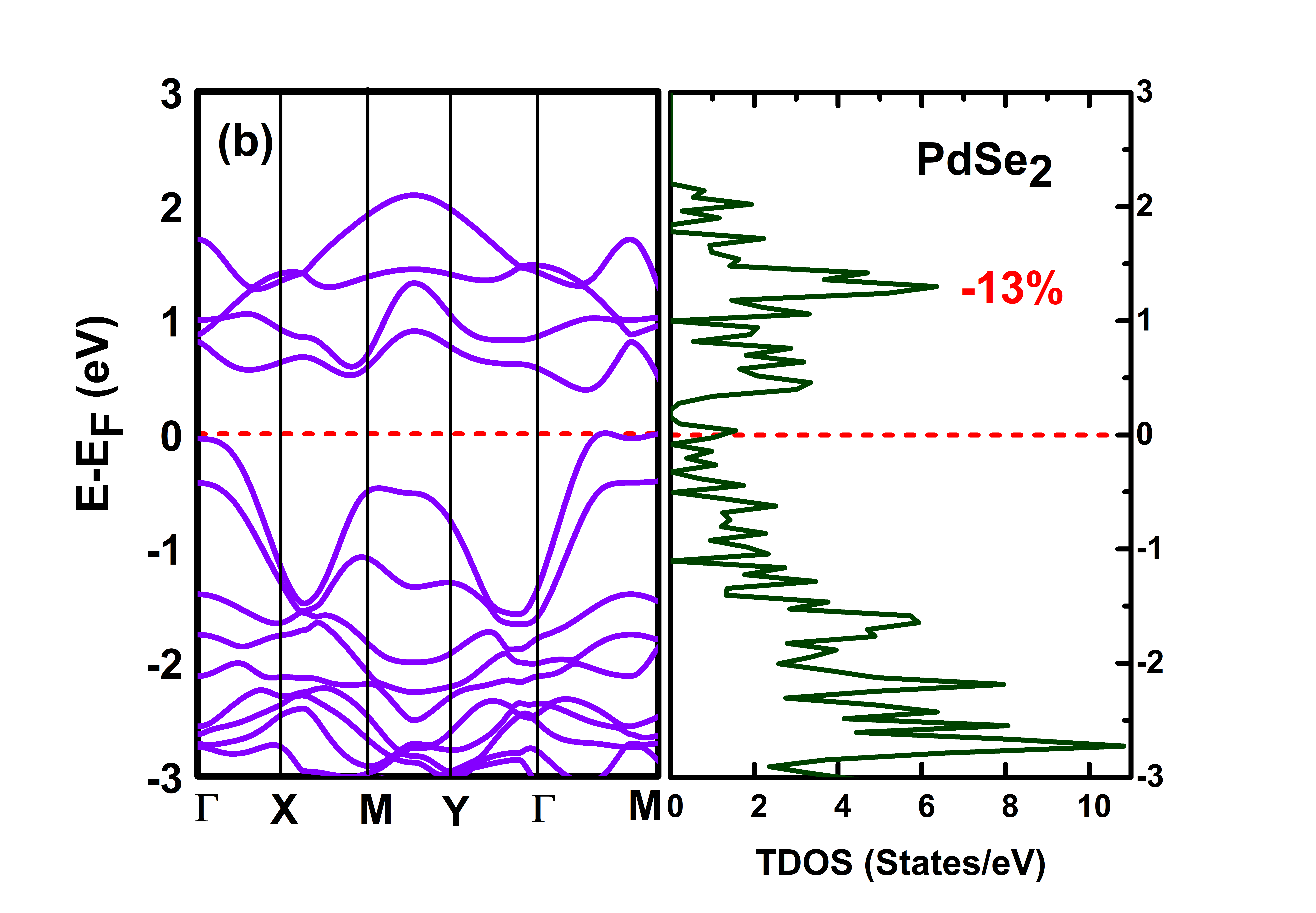 |
| --- | --- |

**Figure S7.** (Colour online) Electronic band structure and total density of states (TDOS) States/eV. (a) For penta-PdSe_2_ monolayer under critical tensile strain (+9%) and (b) For penta-PdSe_2_ monolayer under critical compressive strain (-13%).


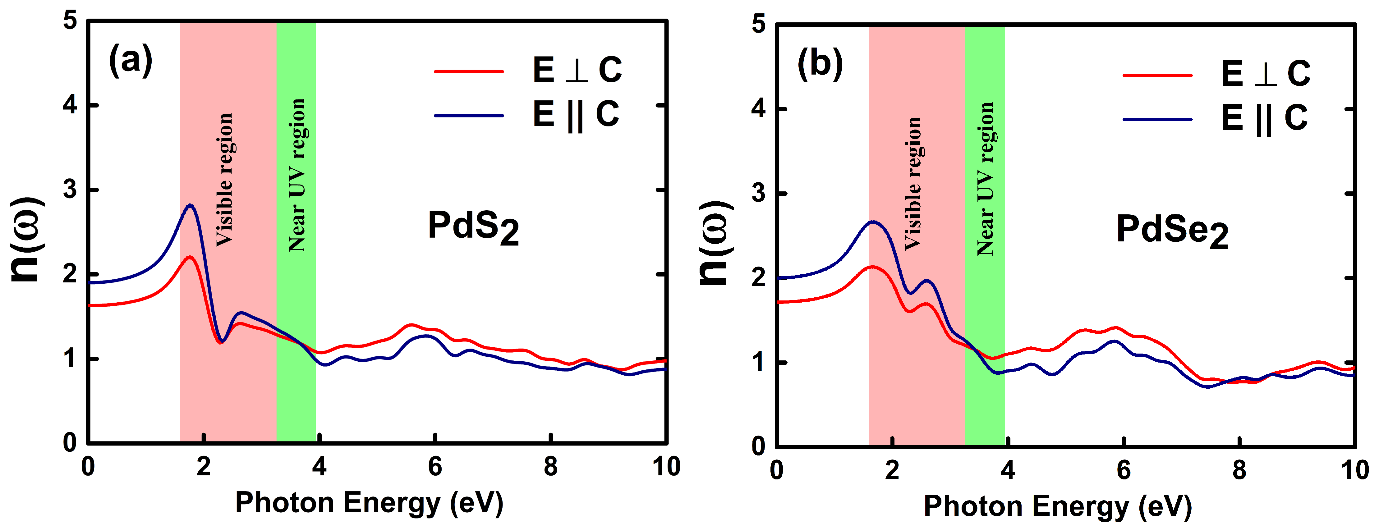


**Figure S8.** (Colour online) Refractive index $n(\omega)$ of penta-PdQ_2_ monolayers for parallel and perpendicular polarization. (a) For penta-PdS_2_ monolayer and (b) For penta-PdSe_2_ monolayer.


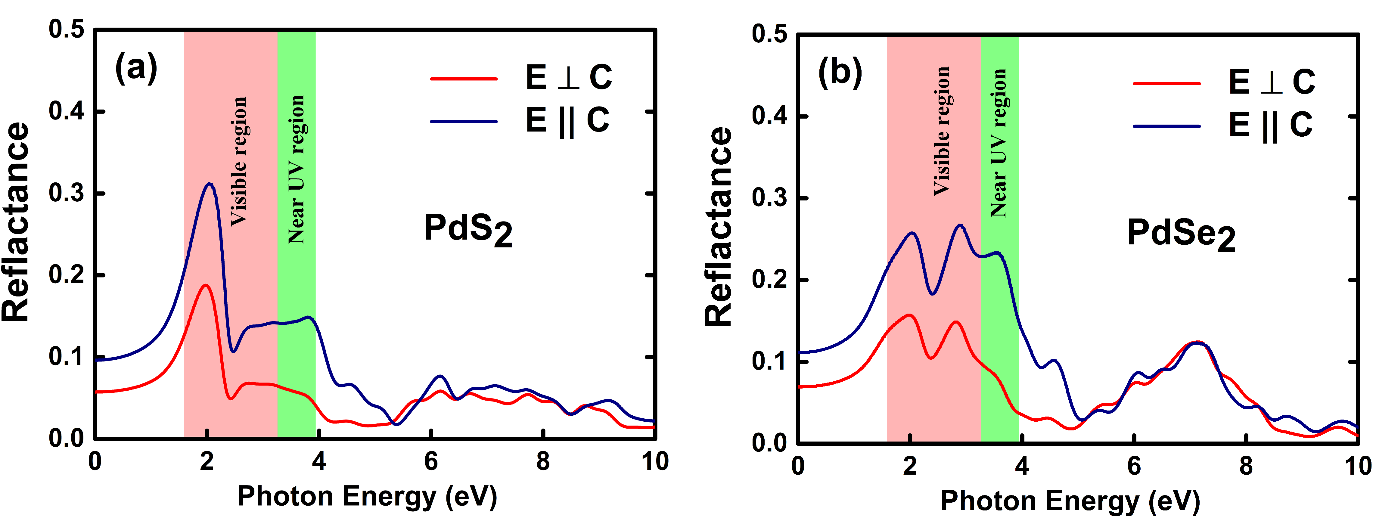


**Figure S9.** (Colour online) Reflectance $R (\omega)$ of penta-PdQ_2_ monolayer for parallel and perpendicular polarization. (a) For penta-PdS_2_ monolayer and (b) For penta-PdSe_2_ monolayer.

| **Penta-PdS_2_ Monolayer** | | | | **Penta-PdSe_2_ Monolayer** | | | |
| --- | --- | --- | --- | --- | --- | --- | --- |
| Compressive strain %  (-Ve) | | Tensile strain % (+Ve) | | Compressive strain % (-Ve) | | Tensile strain % (+Ve) | |
| -13 % | -345.00 cm^-1^ | -- | -- | -13 % | -1110 cm^-1^ | -- | -- |
| -12 % | -123.81 cm^-1^ | -- | -- | -12 % | -1635 cm^-1^ | -- | -- |
| -11 % | -62.14 cm^-1^ | -- | -- | -11 % | -76.71 cm^-1^ | -- | -- |
| -10 % | -56.78 cm^-1^ | -- | -- | -10 % | -89.43 cm^-1^ | -- | -- |
| -9 % | -7.35 cm^-1^ | -- | -- | -9 % | -78.63 cm^-1^ | +9 % | -23.11 cm^-1^ |
| -8 % | -2.30 cm^-1^ | -- | -- | -8 % | -63.03 cm^-1^ | +8 % | -6.0 cm^-1^ |
| -7.0 % | -57.47 cm^-1^ | +7.0 % | Positive Frequency | -7.0 % | Positive Freq. | +7 % | Positive  Frequency |
| -6.0 % | -8.05 cm^-1^ | +6.0 % | Positive Frequency | -6.0 % | -65.78 cm^-1^ | +6 % | -12.2 cm^-1^ |
| -5.0 % | Positive  Freq. | +5.0 % | Positive Frequency | -5.0 % | -65.13 cm^-1^ | +5.0 % | -40.49 cm^-1^ |
| -4.0 % | -23.15 cm^-1^ | +4.0 % | Positive Frequency | -4.0 % | -37.65 cm^-1^ | +4.0 % | Positive Frequency |
| -3.0 % | -48.68 cm^-1^ | +3.0 % | Positive  Frequency | -3.0 % | -32.52 cm^-1^ | +3.0 % | Positive  Frequency |
| -2.0 % | Positive Freq. | +2.0 % | Positive  Frequency | -2.0 % | -35.62 cm^-1^ | +2.0 % | -7.50 cm^-1^ |
| -1.0 % | Positive Freq. | +1.0 % | -61.84 cm^-1^ | -1.0 % | -50.80 cm^-1^ | +1.0 % | Positive  Frequency |

**Table S1**. Phonon frequencies (cm^-1^) at Γ-point for different Compressive (-Ve) and tensile (+Ve) strain % on 2D penta-PdQ_2_ monolayers.
